# Motor cortex flexibly deploys a high-dimensional repertoire of subskills

**DOI:** 10.1101/2025.09.07.674717

**Published:** 2025-09-08

**Authors:** Elom A. Amematsro, Eric M. Trautmann, Najja J. Marshall, L.F. Abbott, Michael N. Shadlen, Daniel M. Wolpert, Mark M. Churchland

**Affiliations:** 1 Department of Neuroscience, Columbia University Medical Center, New York, NY, USA.; 2 Zuckerman Mind Brain Behavior Institute, Columbia University, New York, NY, USA.; 3 Center for Theoretical Neuroscience, Columbia University Medical Center, New York, NY, USA.; 4 Kavli Institute for Brain Science, Columbia University, New York, NY, USA.; 5 Grossman Center for the Statistics of Mind, Columbia University, New York, NY, USA.; 6 Howard Hughes Medical Institute, Columbia University, New York, NY, USA.; 7 Computational and Biological Learning Lab, Department of Engineering, University of Cambridge, Cambridge, United Kingdom.

## Abstract

Skilled movement often requires flexibly combining multiple subskills, each requiring dedicated control strategies and underlying computations. How the motor system achieves such versatility remains unclear. Using high-density Neuropixels recordings from primary motor cortex (M1) in macaques performing a challenging force-tracking task, we reveal that M1 activity is much higher-dimensional, and far more flexible, than traditionally assumed. Although our task employed only a single external degree of freedom, neural dynamics reflected transitions amongst many dimensions and multiple distinct computations. Different behavioral control strategies were associated with distinct neural locations and dimensions, sometimes used compositionally. Groups of population-level factors became active when a particular form of dynamics was needed, and remained silent otherwise. Neural activity was thus dominated by the engaged subskill, and could be very different even for matched motor output. These findings challenge prevailing views of M1, and reveal an unexpectedly flexible and high-dimensional neural system underlying skilled motor behavior.

## Introduction

Competing in Indonesia, the professional surfer Stephanie Gilmore scored a perfect ten by linking bottom turns, off-the-lip maneuvers and tube-riding into a single fluid ride. Her accomplishment illustrates that motor control often requires composition: in her case, flexibly switching, and even simultaneously blending, multiple surfing subskills. Might repertoires of subskill-specific motor computations ^1^ be instantiated and flexibly deployed by primary motor cortex (M1), the brain area most responsible for skilled voluntary movement? While this ‘flexible-repertoire hypothesis’ is appealing, past and current perspectives suggest the opposite: M1 activity appears constrained by relatively few degrees of freedom ^2^, with a repertoire of available patterns that is fixed and small ^3,4^ rather than flexible and large. A fixed computation would accord with M1’s status as a primary cortical area, one synapse from spinal motoneurons. Indeed, a venerable assumption is that task manipulations can disambiguate hypotheses by providing different views of a consistent M1 computation ^5,6^. Alternatively, M1’s apparent inflexibility may reflect experimental limitations: laboratory tasks are rarely designed to engage many subskills, and the scale of neural recordings may have been insufficient to reveal signatures of computational flexibility.

**Figure 1.**
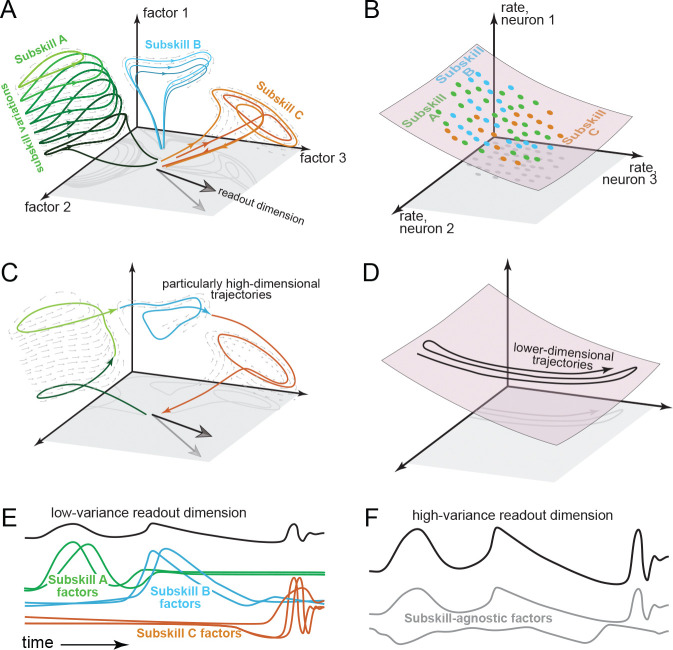
Flexible-repertoire and constrained-manifold predictions. Left column: flexible-repertoire hypothesis; right column: constrained-manifold hypothesis. (**A**) The flexible-repertoire hypothesis predicts high-dimensional activity, resulting from multiple subskill-specific computations that use dedicated locations, dimensions, and dynamics. (**B**) Alternatively, activity occupies a low-dimensional manifold (dots indicate visited states), reused across subskills (colors). (**C**) Flexible-repertoire predictions relevant to the present task. When a task requires multiple subskills, activity should be high-dimensional even if motor output is simple. Some conditions may compositionally reuse subskills, yielding changing locations and dimensions. (**D**) If a task uses one physical dimension and few independent parameters, constrained-manifold activity should become lower-dimensional still. (**E**) Motor output is predicted to rely on different factors at different moments. Consequently, the readout dimension captures little population variance. (**F**) Traditionally, motor readouts derive from a few continuously active high-variance signals.

Those potential signatures are suggested by a burgeoning interest in multi-task recurrent networks. In recurrent networks, a dynamical flow-field governs a ‘neural state’ ^7,8^ in a state-space where each dimension captures one network factor ^9^. Factors are time-varying activity patterns that form the basis both for network outputs and for ‘internal’ network computations. Consequently, factors also form a basis for single-neuron responses. Multi-task networks leverage multiple forms of internal dynamics, each employing different factors ^10–17^. Flexibility arises from the variety of dynamics and their combinatorial potential. The flexible-repertoire hypothesis proposes M1 may function similarly (Fig. 1A).

Like most prevailing conceptions of M1 ^2–4,18^, the flexible-repertoire hypothesis posits dynamics within a state-space ^8,19–21^. Somewhat unusually, the flexible-repertoire hypothesis proposes a very high-dimensional state-space, with different subskills engaging different regions and dimensions (Fig. 1A). Such separation is necessary to isolate different forms of dynamics, thus preventing interference ^22,23^. The potential to combine subskills simultaneously – perhaps on her next wave, Ms. Gilmore blends maneuvers in a surprising new way – requires neural activity that is not restricted to previously observed regions of state-space. These predictions oppose the prevailing view (Fig. 1B) that firing rates are constrained by a low-dimensional manifold ^2,18,24^, preserved and reused across subskills ^3,4^. Contrasting predictions are clearest if a task employs few external degrees of freedom yet engages multiple subskills. Traditionally, eliminating degrees of freedom can only constrain activity further (Fig. 1D). In contrast, under the flexible-repertoire hypothesis, dimensionality is effectively unbounded; it may grow very high if multiple subskills are engaged across conditions or compositionally within-condition (Fig. 1C). Traditionally, readout dimensions target high-variance signals: a few continuously active factors that resemble motor output (Fig. 1F). Under the flexible-repertoire hypothesis, each readout-dimension is impacted by many factors, none consistently resembling the output. Most neural activity is thus output-null ^9,25^ and readout-dimensions capture little population variance (Fig. 1E).

Some recent findings potentially align with the flexible-repertoire hypothesis. Learning can alter neural degrees of freedom ^26^, high-dimensional assumptions improve decoding ^27^, and population activity contains numerous ‘small’ (low-variance) signals ^28^. Activity can also traverse different dimensions ^23,29–38^: e.g. when switching from walking to lever-pulling, or switching the arm used to reach. However, these situations involve large changes in muscle-muscle correlations, including which muscles are used. Changes in neural dimensions are thus expected under nearly any hypothesis, including simply because motor-output dimensions change (a common and reasonable interpretation).

An ideal test of the flexible-repertoire hypothesis requires the situation outlined above: a challenging task that engages multiple subskills, yet where simple motor output employs one fixed axis. We leveraged the ‘Pac-Man’ force-tracking task ^28^, which employs one degree of freedom (pressing forward) and is isometric: the arm does not move. Essentially all external variables – including muscle activity and cursor position – mirror force or its derivative. Yet accurate performance demands various combinations of reactive (closed-loop) and proactive (open-loop) strategies. The flexible-repertoire hypothesis predicts (i) activity will be high-dimensional despite physical task simplicity, (ii) the neural manifold will not be preserved: activity will occupy different regions and dimensions across subskills, (iii) some conditions will use multiple subskills compositionally, and (iv) each subskill will be reused across some conditions but not others. A final strong prediction (v) is that neural activity should differ when subskills differ, even if motor output is identical.

An ~100-neuron population is inadequate to resolve activity that is predicted to be diverse, temporally rich, and high-dimensional. We thus employed primate-optimized Neuropixels Probes ^39^ to record large (~1000) populations. To aid interpretation via comparison with a downstream neural population, intra-muscular recordings isolated single motor units, each mirroring the spikes of one spinal motoneuron. Motoneuron activity was simple and low-dimensional. M1 activity was high-dimensional and obeyed flexible-repertoire predictions. Thus, the computational role of M1 appears far more flexible than typically envisioned. When one considers the repertoire of abilities acquired in our lifetimes, M1 dimensionality and the variety of dynamics it instantiates are presumably vast.

## Results

### A challenging force-tracking task evokes multiple behavioral strategies

**Figure 2.**
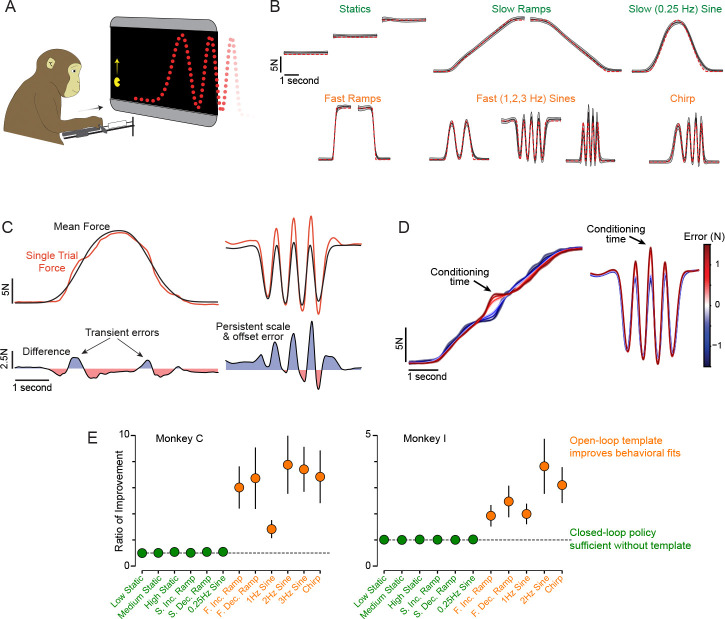
Task and behavior. (**A**) Task schematic. The leftward-scrolling dot-path revealed ~1 second of future target force. (**B**) Target (dashed red) and trial-averaged forces (black). Gray envelopes: across-trial SD. Data for Monkey C (Monkey I; Supp. Fig. 1). (**C**) Example single-trial forces (red), mean force (black) and error (their difference, bottom) during 0.25 and 2 Hz sines. (**D**) Trial-averaged forces (slow-increasing ramp and 2 Hz sine), conditioned on error magnitude (six quantiles) at each trial’s mid-point. Envelopes: SD. Scale bar: error at conditioning time. (**E**) Behavioral-fit improvement when Autoregressive Exogenous Input models (Methods) used profile-specific templates. At each moment in each trial, change-in-force was fit given recent errors and (if used) the template. Fits improved only for some profiles (orange). Bars: SD across partitions, equivalent to SEM.

Two rhesus macaques (monkeys C and I) performed an isometric force-tracking task ^28^. The height of a Pac-Man icon mirrored forward force applied to an immovable handle (Fig. 2A, Supp. Movie 1). The task required Pac-Man’s height to be continuously adjusted to intercept a leftward-scrolling dot-path. Dot-paths specified 12 (monkey C) and 11 (monkey I) distinct target force profiles (i.e. conditions, Fig. 2B).

In a moving multi-joint arm, muscle activity rarely mirrors endpoint force. In contrast, the Pac-Man task is isometric, involves a single degree of freedom (pushing forward), and is performed by elbow and shoulder extensors working together. Muscle activity is thus expected to correlate strongly with endpoint force, as confirmed via motor-unit recordings in Monkey C (see below). Additionally, nearly all ‘external’ parameters reflect force or its derivative. Dot-height specifies desired force, closely tracked by actual force. Pac-Man’s position and velocity mirror force and its derivative. Joint torques will be proportional to force. Stretch-receptor feedback will reflect muscle tension (which reflects force) or its derivative. Under all current hypotheses, activity should become simpler and lower-dimensional; movement is uni-directional, there are few independent parameters, and biomechanical complexity is minimized. Conversely, under the flexible-repertoire hypothesis, neural dimensionality is not bounded by external degrees of freedom but instead reflects the variety of engaged subskills.

Encouraged by a nonlinear reward schedule, monkeys became remarkably adept at matching a wide variety of target profiles (Fig. 2B, gray envelopes show across-trial SD). Observing behavior in real time (Supp. Movie 1) suggests that performing this challenging task involves multiple strategies. When force changes slowly, a reasonable strategy is to focus on reducing the current discrepancy between Pac-Man’s height and dot height (a ‘closed-loop’ strategy). In agreement, errors were transient – i.e. corrected quickly – during low-frequency profiles (Fig. 2C, left). Higher frequencies require recognizing the approaching profile, with force produced proactively using an internal template (an ‘open-loop’ strategy). A mis-scaled or mis-centered template would cause persistent errors (e.g. Fig. 2C, right). To examine error persistence, we conditioned trial-averaged forces upon error in each trial’s middle (Fig. 2D). For slowly changing profiles, error diminished steadily over ~300 ms, consistent with closed-loop tracking. For high-frequency profiles, error disappeared swiftly then reappeared later, consistent with an internal template that almost (but not quite) matched the target. When fitting control-theory models to single-trial behavior, closed-loop strategies sufficed at lower frequencies. Higher frequencies benefited from including open-loop templates (Fig. 2E).

Open-loop strategies must be template-specific, and high-level performance may combine closed- and open-loop strategies. Under the flexible-repertoire hypothesis, this would prompt M1 to switch amongst subskills. For this task, we define a ‘subskill’ to be a computation (implemented by network dynamics) that can generate a particular type of force-profile (e.g. 2 Hz sinusoids of various phases) and/or implement closed-loop corrections. More generally, a subskill is a motor ability suitable to generate muscle-activity patterns ^40^ (including corrections) in some situations but not others ^1^. We accept a likely continuum between completely distinct subskills versus subskill variations; our analyses are designed to respect both possibilities. We also anticipate that subskills might be reused compositionally: e.g. the slow (0.25 Hz) sinusoid might recruit, sequentially, subskills used for slow rising and falling ramps. Rising and falling ramps might in turn use the same or different subskills (control policies might or might not be symmetric). How performance is (or isn’t) segmented into subskills is difficult to infer externally but should, under the flexible-repertoire hypothesis, become apparent in the neural data.

### High-dimensional neural activity shows subskill-specific features

**Figure 3.**
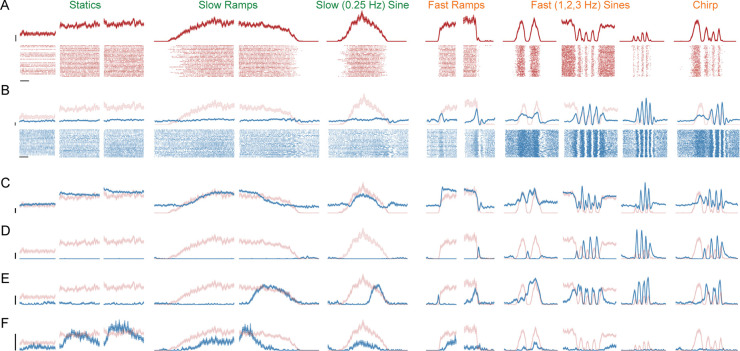
Example motor-unit and M1-neuron responses. (**A**) Trial-averaged firing-rate for an example motor unit. Envelopes indicate SEM. Horizontal and vertical bars denote 500 ms and 5 spikes/sec. Spike rasters are plotted with one line per trial. This mean motor-unit response is repeated below, providing a reference for expectations if a response reflects force. (**B**) Same but for an example M1 neuron. (**C**) Mean rate of an atypical M1 neuron whose response resembles force. (**C-F**) Additional, more typical, example M1 neurons.

Neural activity was recorded primarily using 384-channel NeuroPixels Probes (1.0 NHP ^39^) and pooled across multiple probes and sessions. Testing flexible-repertoire predictions requires a large population of individually well-resolved responses. We thus analyzed only stable isolations with excellent estimates of rate modulation (1257 and 864 neurons for Monkey C and I; additional recordings made for task variants described later). For example, the neuron in Fig. 3C had a firing-rate range 47 times greater than its average SEM (average ratio of 32 for this dataset; envelopes show SEMs). Microstimulation of the recorded region activated the task-relevant muscles: *anterior deltoid* and *triceps brachii*. Motor-unit recordings (134 total) employed these same muscles. Individual motor-unit spikes (corresponding one-to-one with motoneuron spikes) were sorted using customized methods ^28^. Motor-unit activity was relatively simple; each motor unit, if active, correlated with force. The example in Fig. 3A was typical: its response reflects force overall (r = 0.87) and within-condition (range 0.58–0.97). Motor-unit responses were not identical ^28^, but all correlated with force when active (mean *r* = 0.79). Consequently, the first principal component (PC) of motor-unit activity correlated strongly with endpoint force (*r* = 0.85).

The flexible-repertoire hypothesis predicts that, despite external task simplicity, M1 activity should involve many factors and thus be high-dimensional. In agreement, accounting for 90% of population variance required >75 PCs (both monkeys), versus 4 for the motor units. M1 dimensionality in reaching tasks is often estimated as ~10–12 ^2,4^. Studies ^41–43^ have stressed that dimensionality exceeds that predicted by representational models, but dimensionality in the present task is higher still, despite it using only one direction.

High-fidelity firing-rate estimates make it simple to appreciate, through inspection, the reasons for high-dimensionality. A few neurons (e.g. Fig. 3C) had responses that consistently resembled force, yet this was very rare (Supp. Fig. 2). For example, the neuron in Fig. 3B mirrors force only when force changes rapidly. The neuron in Fig. 3D responds out of phase with force, at only some frequencies. The neuron in Fig. 3E tracks force during only some portions of some conditions. The neuron in Fig. 3F responds only during low-frequency forces. These examples illustrate a common source of diversity: putatively subskill-specific features. A neuron could be active for some conditions (or some parts of some conditions) and inactive for others, despite similar force ranges. More generally, neurons typically had activity whose relationship with force changed across conditions. The combination of between- and within-condition response variety yielded extremely diverse responses that defy easy explanation under any traditional hypothesis. For example, single-neuron activity has often been observed to reflect muscle force ^6,44–46^ and/or related parameters. Given traditional expectations, that relationship should be particularly straightforward in this task; forces are unidirectional and there are few other independent parameters. Instead, correlations with force (and its derivative) were generally very small (Supp. Fig. 2). Nevertheless, force could be accurately decoded (see below). Understanding this seeming paradox requires stepping through flexible-repertoire predictions.

### Neural state-space locations form a map of subskills

**Figure 4.**
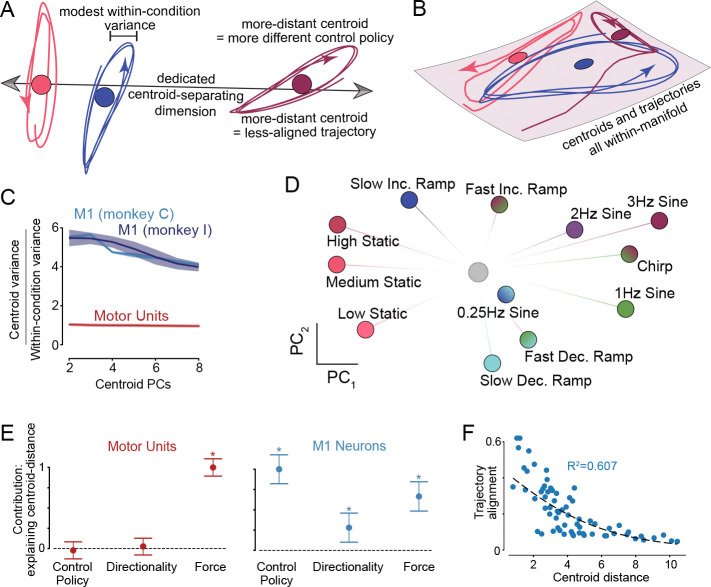
State-space location reflects subskill. (**A**) Flexible-repertoire predictions regarding each condition’s activity centroid (mean activity, colored circles) and its within-condition trajectory (colored traces). The hypothesis predicts dimensions (arrow) that preferentially separate centroids versus capturing trajectories. More-distant centroids should correspond to less-similar subskills and less-aligned trajectories. (**B**) Constrained-manifold predictions. Across conditions, trajectories may have different means, but these simply occur in the same manifold. (**C**) Ratio of centroid-variance to within-trajectory-variance (both un-normalized) captured by centroid PCs. Ratios >1 indicate centroid-separating dimensions differ from those that best-capture trajectories (as in A). Ratios near unity accord with panel B. Envelopes: SD of bootstrapped values (equivalent to SEM). (**D**) Organization of M1 centroids in their top PCs. Gray dot indicates baseline activity. Color choices reflect the factors engaged for that condition (assessed below). Lines connecting baseline and centroids added for visualization. Data for Monkey C. (Monkey I; Supp. Fig. 3). (**E**) Contribution of behavioral distances when fitting centroid distances. Significance (*, *p* < 0.005, two-sided) and 95% CIs computed via each co-efficient’s t-statistic. Contributions are normalized (maximum one). (**F**) Trajectory alignment versus centroid-distance. One dot per condition-pair. Fit via logistic model (*p* < 10^−5^). Monkey I analysis and additional details in Supp. Fig. 3.

Noise-robust network dynamics require low trajectory-tangling: nearby states should avoid moving in different directions ^22,23^. Under the flexible-repertoire hypothesis, subskills are proposed to leverage different dynamical flow-fields. Low tangling thus requires separation of subskills into different state-space regions (Fig. 1A). When considering where activity is centered for each condition (its ‘centroid’), four predictions follow (Fig. 4A). First, to prevent overlapping / tangled trajectories, centroids should be separated in dimensions beyond those capturing within-condition trajectories. Second, centroids should be organized, with less-similar subskills having more-distant centroids (the more flow-fields differ, the greater the separation needed). Third, more-distant centroids should correspond to trajectories in less-aligned dimensions (separating dynamics into different dimensions also reduces tangling ^23^). Finally, trajectory tangling should remain low when computed across all conditions. A constrained-manifold makes different predictions (Fig. 4B): conditions may have differing means – e.g. due to differing mean forces – but these differences don’t reflect subskill *per se* (subskills reuse the same manifold), and simply occur in the same space as the trajectories.

We computed each condition’s state-space trajectory, then estimated its centroid by averaging over time. For now we ignore the possibility of compositionality (considered later) and treat each condition as involving one potential subskill. M1 populations obeyed flexible-repertoire predictions: activity was separated in dimensions that preferentially captured centroid distances versus within-condition trajectories (Fig. 4C, blue). In contrast, the space capturing motor-unit centroids was merely the same space that captured the trajectories themselves (Fig. 4C, red). M1-centroid organization (Fig. 4D) reflected features beyond motor output. The three static centroids lay near each other, despite mean forces being very different. Increasing- and decreasing-ramp centroids lay far apart (top and bottom) despite nearly identical mean force. Conditions involving closed-loop versus open-loop strategies had particularly distant centroids.

To explore this organization, we modeled centroid distances (between all pairs, in full-dimensional space) as a linear function of three behavioral quantities: difference in mean force (force distance), the degree to which force increased versus decreased (directionality distance), and how well a control-policy model generalized from one condition to another (control-policy distance, see Methods). Force-distance should matter under nearly any hypothesis, and thus be predictive for both M1 and motor-unit populations. Directionality and control-policy distances relate to the subskill used. These distances should be irrelevant for the motor-units but should matter (under the flexible-repertoire hypothesis) for M1. Centroid-distances were fit well for both motor-units (*R*^2^ = 0.86) and M1 (*R*^2^ = 0.808, *R*^2^ = 0.691; Monkey C and I; Supp. Fig. 4). Motor-unit fits relied solely on force distance (Fig. 4E, red). All three distances were explanatory for M1, and control-policy distance was most explanatory (Fig. 4E, blue). Also as predicted, more-distant M1 centroids involved trajectories in less-aligned dimensions (Fig. 4F; alignment index described below). Thus, a substantial portion of M1 activity reflects subskill-organization rather than motor output. Also as predicted, trajectory tangling was far lower (16%) for M1 populations versus motor-units.

### Neural state-space dimensions are subskill-specific

We used the subspace-alignment index ^29^ to assess whether activity shares dimensions across condition-groups. Groups were based on force-profile features: e.g. all statics, or all fast sines. All groups included a similar range of forces and (except for statics) both increasing and decreasing forces. The alignment index ranges from zero (subspaces share no dimensions) to one (all shared). For a constrained manifold, alignment should approach unity. The flexible-repertoire hypothesis predicts varied alignment: high when two condition-groups share subskills and low otherwise. For the motor-units, alignment was consistently high (mean 0.85; >0.75 every comparison; Supp. Fig. 7). M1 populations had variable alignment (0.14–0.81 monkey C; 0.33–0.81 monkey I) even though all condition-groups involved force in the same direction. Low alignment occurred for condition-groups that likely used different control strategies: e.g. statics versus fastsines (0.14 and 0.35, monkey C and I). Overall, condition-groups tended to share only half their dimensions: alignment averaged 0.41 and 0.51 (monkey C and I; also see Supp. Fig. 5, Supp. Fig. 6, Supp. Fig. 7).

Why do these results differ from Gallego et al. ^4^, who found strong alignment (>0.8) across sub-tasks, or Golub et al. ^3^, who found preserved covariance? The flexible-repertoire hypothesis predicts high alignment when situations reuse subskills, which seems likely in these prior tasks. In agreement, we also observed examples of reuse: e.g. the slow sine and slow-ramp group were well-aligned (0.81 for both monkeys), unlike fast-sine and slow-ramp groups (0.36 and 0.44). Under the hypothesis of reuse, activity during the rising phase of the slow sine should overlap dimensions for the slow-rising ramp, and come closest to that centroid. During the falling phase, activity should overlap dimensions for the slow-falling ramp, and come closest to that centroid. This was confirmed, along with additional forms of reuse (Supp. Fig. 9, Supp. Fig. 10).

### Population factors reflect selective recruitment of subskill-specific dynamics

Under the flexible-subspace hypothesis, M1 expresses a rich variety of subskill-specific dynamics, each employing a group of factors. There should thus be many total factors spanning many total dimensions. Each factor-group is predicted to be sparsely active, becoming engaged only when that subskill is used (Fig. 1E) and generating (at those moments but not others) a basis for outgoing commands. Large-scale recordings should allow direct tests of these predictions, via examination of estimated factors, if one can bypass limitations of standard factor-estimation methods. For instance, PCA tends to combine sparsely active factors, something traditionally overcome through supervision: guessing which conditions or times might use different factors ^29^. To avoid such guessing and allow potential subskill-divisions to emerge directly from the data, we employed a novel unsupervised method, Sparse Component Analysis (SCA) ^47^. SCA behaves much like PCA (and captures similar overall variance: 94% as much for both monkeys), but avoids PCA’s tendency to concentrate variance in its first components and thus mix sparsely active factors. If factors are not sparse, SCA cannot make them so, nor does it impose assumptions regarding the nature of potential sparsity. Under the constrained-manifold view, SCA should thus identify relatively few factors, most active at most times. Under the flexible-repertoire hypothesis, SCA should identify many (and thus individually low-variance) factors, with different factor-groups active at different moments. Yet sparsity should not be too extreme; factor-groups should be reused when different moments can leverage the same subskill.

**Figure 5.**
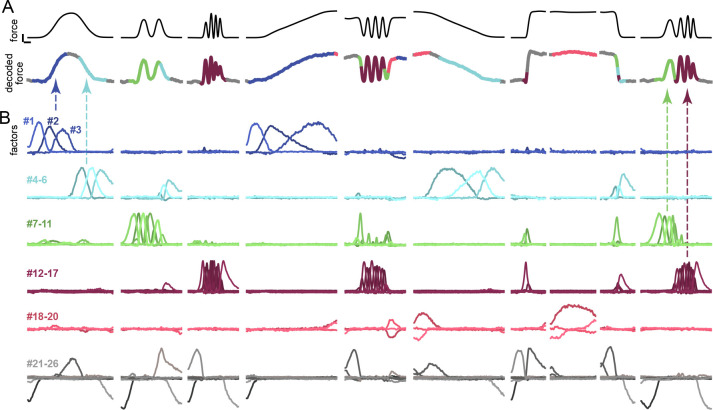
Factors are numerous and make temporally-specific contributions. (**A**) Actual and decoded force (a weighted sum of factors below) across conditions. Trace color reflects which factor group contributed most to decoding at that moment. (**B**) Each factor is a projection of the population response onto an SCA-identified dimension. Factors form rough groups, indicated by color. Arrows illustrate two conditions where decoded force depended upon sequentially active factor groups. Data from Monkey C (Monkey I; Supp. Fig. 11). Not shown are a few factors dominated by sampling error (when many SCA factors are requested, any ‘extra’ factors typically just capture noise).

SCA identified many factors (Fig. 5B), which formed rough groups. A ‘blue’ factor-group was engaged only when force rose slowly: during the 0.25-Hz sine and slow rising ramp. A ‘cyan’ factor-group became engaged when force decreased slowly. ‘Green’ and ‘purple’ groups were engaged during medium-frequency (1–2 Hz) and high-frequency (2–3 Hz) sinusoids. A ‘red’ group was engaged when high force was maintained. A ‘gray’ group tended to reflect transitions: e.g. the darkest gray factor increased before each transition from rest. Force was accurately decoded from these factors (Fig. 5A, *R*^2^ = 0.98), much as it was from the full population (cross-validated analysis in Supp. Fig. 16). Different factor-groups contributed at different moments (reflected by decoded-trace color, Fig. 5A). No factor was dominant; each contributed 1.1–6.3% of total captured variance. A factor that accounts for 2% of population structure would traditionally have been considered irrelevant (and would have been difficult to resolve in any case). Yet these low-variance factors made large contributions at key moments. For example, artificially silencing the blue factor-group impaired decoding of slowly increasing forces. Dynamics differed across conditions (Supp. Fig. 12), reflecting the active factors; e.g. 1 Hz and 3 Hz sines involved medium- and high-frequency oscillatory population dynamics, reflecting (respectively) green and purple factor-group activity.

SCA cannot ‘invent’ unrepresentative sparse factors ^47^. Doing so would significantly increase reconstruction error, something discouraged by the objective function (Methods) and absent empirically (Supp. Fig. 15). Two further observations illustrate this point. First, motor-unit SCA factors were not sparse (Supp. Fig. 14), even when sparsity was encouraged at the expense of increased reconstruction error (Supp. Fig. 15). Second, M1 SCA factors were in fact very representative of single-neuron response features. M1 responses were extremely diverse, consistent with numerous factors. Some neurons were sparsely active, just like the factors. More generally, neurons displayed condition-dependent relationships with force, as expected if a neuron reflects multiple sparsely active factors (which was indeed common; Supp. Fig. 13). Factor activity also accords with subspace-alignment results (discussed below). Thus, SCA simply reveals, in greater detail and without supervision, effects visible in other ways.

The presence of many sparsely active factors upends some common expectations. For example, in a ‘force-centric’ task, one would traditionally expect force to be strongly represented. And in one sense it was: a force-readout dimension yielded accurate decoding (cross-validated *R*^2^ = 0.99 and 0.96; Monkey C and I). However, across different degrees of regularization, this dimension captured at most 13% and 7% of population variance (Supp. Fig. 16). This seeming paradox is resolved by the observation that (as predicted in Fig. 1E) force decoding relied on many transiently active factors. In contrast, the motor-unit force-readout dimension captured up to 73% of population variance (Supp. Fig. 16) and relied on one continuously active factor (Supp. Fig. 14). Thus, force was encoded in a traditional manner by the motor units, and in a very different way by the M1 population.

### Subksills are reused compositionally across conditions

M1 activity is unexpectedly sparse, yet less sparse than it could be; neither M1 factors nor M1 neurons approached the extreme sparsity found in songbird area HVC ^48^. Factors were often active for sizable spans of time, and were reused in new combinations across conditions. Arrows (Fig. 5) highlight two examples of sequential reuse. The 0.25 Hz sine engages blue then cyan factor-groups, also engaged (respectively) during slow increasing and decreasing ramps. The chirp engages green then purple factor-groups, also engaged (respectively) during 1–2 Hz and 2–3 Hz sines. Fast ramps recruit green and purple groups simultaneously (plus the cyan group for the decreasing ramp), perhaps reflecting the ramp’s broad frequency content. The oscillatory dynamics of green and purple factor-groups, patent during 1–3 Hz sines, are less-obvious during fast ramps yet still visible in the eigenvalue spectra of dynamical fits (Supp. Fig. 12H), reminiscent of what occurs during swift reaches ^19,49^. Some factor combinations (e.g. coactive cyan, green and purple groups) were specific to one condition (e.g. fast-decreasing ramps).

The scale of our recordings allowed us to estimate the timecourse of ~26 (monkey C) and ~24 (monkey I) factors. (There may, of course, have been more active factors than we could accurately resolve). Each condition engaged only a subset of these factors: 7.3 on average (range 1–14) for monkey C and 6.3 on average (range 3–9) for monkey I. Accordingly, a given factor was engaged during only some conditions: on average 3.0 of 12 (monkey C; range 1–6) and 4.9 of 11 (monkey I; range 2–7). These findings accord with subspace-alignment results above: conditions typically shared only some dimensions (each factor employs one dimension). Conditions likely to employ multiple subskills engaged more factors: e.g. the chirp engaged 14 and 9 (monkey C and I). Simpler conditions engaged fewer: the high static engaged 2 and 4 respectively. Factor activity replicates (and explains) reuse of dimensions between some conditions but not others (Supp. Fig. 9, Supp. Fig. 10). For example, during the chirp, sequential use of green and purple factors explains the progressive alignment with dimensions used during 1, 2 and 3 Hz sines. Conversely, alignment was low when conditions did not share engaged factors.

### The same motor output can be subserved by different subskills

**Figure 6.**
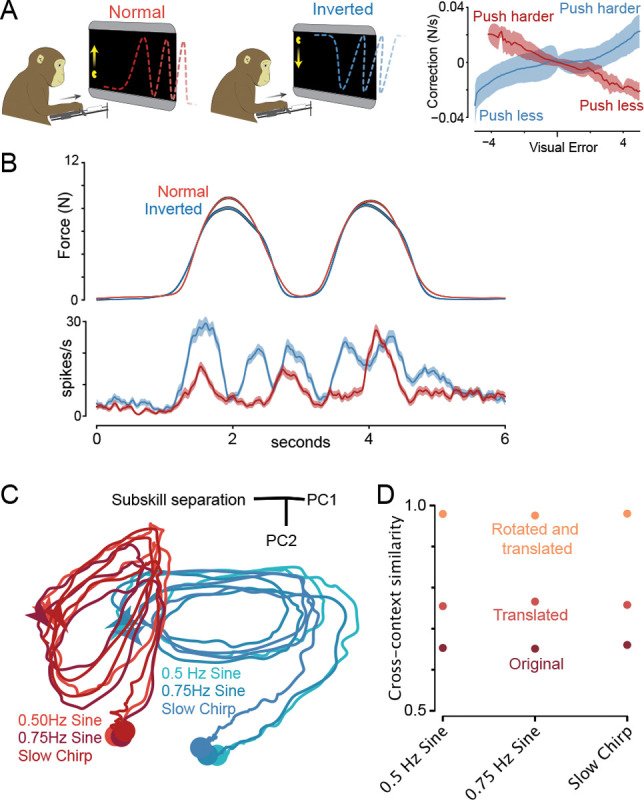
Neural Activity during Normal and Inverted Subskills. (**A**) Left: Task schematic. Right: Change in force’s derivative (±SD) 150 ms after visual error (Pac-Man’s height minus target height). Data for Monkey C (Monkey I: Supp. Fig. 20). (**B**) Trial-averaged force (top) and example-neuron activity (bottom) during a 0.5 Hz sine, for normal and inverted subskills. Envelopes show SEM. To be representative, the example neuron was chosen so that its between-subskill activity difference was near the population median. (**C**) State-space projections of neural activity. Two axes were found via PCA. The third captured between-subskill differences. (**D**) Cross-context similarity of the population response (Methods) between normal and inverted subskills. Before computing similarity, data underwent no transformation (maroon), a similarity-maximizing translation (red-orange), or a similarity-maximizing rotation and translation (orange). Analysis used a relatively high (30) dimensional space to ensure trajectory differences were not lost.

The results above indicate that M1 activity strongly reflects subskill, even more so than motor output. Why was this large effect not previously apparent? One likely reason is that, even when an experiment uses two sub-tasks (e.g. variants of center-out reaching ^4^), they may reuse subskills, yielding a conserved manifold. At the other extreme, for the few prior tasks that surely engaged different subskills (e.g. walking versus lever-pulling ^30^), the axes of motor output differed dramatically. Neural activity is thus guaranteed to differ under any hypothesis, including engaging different output-dimensions within a low-dimensional manifold. The Pac-Man task sidesteps this interpretational hurdle by keeping force direction constant. An even more stringent test would compare neural activity across subskills with matched time-varying outputs. We thus trained monkeys to perform an ‘inverted’ Pac-Man task, in addition to the normal version (Fig. 6A). The inverted task used blue dot paths, with Pac-Man moving downward (from zero force at the top) with increasing forward force. By also inverting visual target profiles, we created condition-pairs with matched forces (Fig. 6B; Supp. Fig. 19, Supp. Fig. 20).

Visuomotor reversals are challenging during continuous-tracking tasks ^1,50^; they cannot be solved by rapid adaptation nor by purely cognitive strategies. Consequently, extensive practice was required to accurately perform both versions in interleaved trials. Post-training, rapid responses to small errors had opposing signs (Fig. 6A), confirming subskill-specific control policies. An intriguing possibility is that two related but distinct internal subskills may develop within M1. Each subskill’s trajectory would be a copy of the other, and they would produce the same typical motor output, yet they would unfold in different locations and dimensions. In contrast, if changing motor output ^30,35,37^ is necessary to cause changes in M1 neural dimensions / locations, then neural activity should be nearly identical across these subskills.

Single-neuron responses differed across subskills, despite matched forces (Fig. 6B, further examples and analyses in Supp. Fig. 19, Supp. Fig. 20, Supp. Fig. 23). Neural trajectories shared similar shapes across subskills (Fig. 6C) and could be brought into almost perfect register via translation and rotation (Fig. 6D; Supp. Fig. 21). Yet trajectories were centered in different locations and occupied different (but not orthogonal) dimensions. Between-subskill trajectory differences were sizable relative to across-force-profile differences (the three similar-colored trajectories). A direct impact of visual features (e.g. dot color) on M1 activity cannot be ruled out, but is unlikely; e.g. we previously found preserved neural activity when different visual cues prompted the same reaches ^51^.

### Different motor outputs can be subserved by the same subskill

Force was closely, but not perfectly, matched across normal and inverted subskills. We performed an additional experiment and set of recordings, in monkey C, to control for these small differences. This experiment also further tests the flexible-repertoire prediction that activity should be influenced more by subskill differences than by motor-output differences. To create a scenario where output differences exceed sub-skill differences, the gain linking force and Pac-Man’s height was incrementally decreased within a session, eventually requiring 67% more force to produce the same height (Supp. Fig. 22). Adaptation to each gain decrement was essentially immediate, consistent with swift alteration of an existing subskill rather than gradual learning of a new one ^1,52^.

Changes in neural activity were much smaller than between normal and inverted Pac-Man, despite far larger changes in force (Supp. Fig. 23, Supp. Fig. 24). The flexible-repertoire hypothesis predicts that, for both large (across-subskill) and modest (during adaptation) changes in activity, motor output should be consistently decodable via a stable low-variance dimension. This was confirmed: *R*^2^ = 0.96 and 0.97 (monkey C and I) when decoding across normal and inverted subskills, and *R*^2^ = 0.96 across gain changes. Readout dimensions captured modest population variance: at most 8.7, 5.0, and 11.7% respectively.

## Discussion

Our findings confirmed the predictions made by the flexible-repertoire hypothesis. They thus argue that M1 instantiates a repertoire of learned computations – one that may be vast when considering our full behavioral repertoires. This perspective evokes a very different picture regarding the nature and purpose of movement-related cortical activity. Its dominant features don’t reflect motor commands, movement parameters, or target properties as has often been assumed, but instead reflect the currently engaged subskill. Rather than being constrained to a low-dimensional manifold, activity moves flexibly amongst many locations and dimensions. Different experimental manipulations cannot be presumed (as in many classic experiments ^5,6^) to provide different views of a consistent M1 computation or representation; they may instead recruit very different computations. This flexibility is surprising for a primary cortical area that communicates directly with the spinal cord. By analogy with primary visual cortex, one might expect M1 to perform a relatively fixed computation. Instead, our data argue that M1 instantiates many acquired computations, organized within a very high-dimensional activity space. These findings speak to a burgeoning interest in the computational basis of motor and cognitive flexibility ^10–16,53–57^. It appears increasingly likely that understanding intelligent behavior requires understanding how neural networks instantiate large repertoires of computations and navigate amongst them to meet the needs of the moment. Our results reveal that this occurs in motor cortex, and document its empirical signatures. These findings illustrate the kind of computational flexibility that our brains possess, and that our field’s theories and hypotheses should attempt to explain. This is not to deny that many computations – e.g. keeping track of heading direction, or maintaining eye position – employ fixed neural manifolds to robustly perform a consistent computation. Yet if a ‘low-level’ primary area such as M1 is flexible, then flexibility may be more the rule than the exception.

The flexible-repertoire hypothesis integrates ideas regarding network dynamics ^9,49,58–60^, subspaces ^18,61–63^, and optimal feedback control ^64–67^. To instantiate different subskills, including subskill-specific control policies ^14,68^, a network must employ a variety of dynamics. This can be accomplished by using different locations and subspaces ^23,36,37^, arranged in a neural space where most dimensions are output-null ^9^. The proposal that distinct M1 subspaces can support distinct computations was initially motivated by orthogonality of preparatory and execution subspaces ^29^. There have since been multiple examples of situation-specific neural dimensions ^23,30–37^ (sometimes accompanied by changes in state-space location ^23,35,37^). Interpretations have varied regarding whether this is more likely to reflect different computations versus motor outputs. Motor output is surely sometimes a key factor ^30,35^, but computation was also deemed potentially relevant: low trajectory tangling requires isolating different dynamics via location and dimensions ^9,22,23^. Our results confirm that different subskills are, on their own, sufficient to cause neural activity to change locations and dimensions. This is true even when motor-output dimensions do not change – indeed, even when motor-output is continuously matched. Furthermore, different neural locations can provide not only variants of the same dynamics (as in ^23,37^), but qualitatively different dynamics and/or control policies.

Synthesizing prior ideas is critical to explain these results – each on its own is insufficient. As one example, the feedback-control model of Kalidindi et al. ^69^ uses limited neural dynamics and incorrectly predicts low-dimensional activity dominated by muscle commands and proprioception. However, their control-centric focus becomes compatible with present results – indeed it helps explain them – if one assumes that rich dynamics instantiate many subskill-specific control policies. As another example, although our results are incompatible with a low-dimensional manifold, that idea remains useful within-subskill. Each subskill’s flow-field presumably constrains how local trajectories unfold ^20,27^. Suppose only one existing subskill is appropriate to the current task. Neural activity would indeed be constrained until learning alters that subskill ^26^. Similarly, low-dimensional network solutions (created via construction ^70^ or training ^59^) often provide good matches with empirical data ^63^. Indeed, encouraging lower-dimensionality can be essential for realistic solutions ^49^. Such networks presumably remain good models of within-subskill dynamics. That said, it is not simply that the flexible-repertoire hypothesis proposes a much higher-dimensional (and less-linear) manifold. By design, the combinatorial expressivity of compositionality – especially the ability to superimpose or blend subskills – will always be capable of generating activity outside a previously observed manifold.

It was recently shown that force-field adaptation shifts activity (in M1 and premotor cortex) during preparation but not movement ^71^. Additionally, new stimulus-movement associations alter preparatory (but not movement) dimensions in mouse premotor cortex ^38^. These findings presumably relate to present results, but in the domain of preparation rather than movement. Thus, the principle of instantiating different learned computations in different locations / dimensions is likely very broad ^9^.

Behavioral investigations indicate normative principles governing how subskills are organized and updated ^1,72–74^. A common metaphor is a landscape containing skills, subskills, and their variants. This landscape is navigated by cognitive-motor processes to produce the variety of actions needed to move within a complex world ^75^. Combined with findings regarding movement variants ^23,37^, our results suggest this conceptual landscape may be reflected in the literal state-space organization of neural activity. This raises intriguing possibilities. Might a subskill’s neural scaffolding be replicated, in a nearby location, when learning a new version of that subskill (a possibility raised by normal-and-inverted Pac-Man results)? Might learning to play squash initially rely on tennis subskills ^1^, with squash-specific subskills ‘split off’ over time? The flexible-repertoire hypothesis makes it possible to pose such questions, and to inquire how our motor repertoires develop and are navigated intelligently by upstream areas.

## Methods

### Subjects and Task

All protocols were in accordance with the National Institutes of Health (NIH) guidelines and approved by the Columbia University Institutional Animal Care and Use Committee (protocol number AC-AABT8672). Subjects were two adult male macaques (Monkeys C and I, Macaca Mulatta), 10 and 11 years-old. During each experiment, the subject sat in a custom-built primate chair with the head restrained via surgical implant and the right arm comfortably secured. To perform the task, subjects grasped a handle with their left hand while resting their left forearm on a small platform that supported the handle. During initial training, subjects developed a stereotyped grasp posture that they found comfortable. To ensure consistent placement within and between sessions, once they achieved this posture we applied tape around the hand and Velcro around the forearm. The handle did not move; isometric force was measured by an attached load cell.

By pressing on the handle, subjects controlled a ‘Pac-Man’ icon, displayed on an LCD monitor (Asus PN PG258Q, 240-Hz refresh, 1,920 × 1,080 pixels) using Psychophysics Toolbox 3.0. The horizontal position of the Pac-Man icon was fixed on the left side of the screen, and its vertical position was directly proportional to the forward force registered by the load cell. A 0 N applied force corresponded to a resting position at the screen’s bottom. Larger forces caused Pac-Man’s position to rise. For the task-variant where we altered the gain (Supp. Fig. 22), the relationship between vertical movement and force was reduced (from the normal gain of 1) to 0.8 and then to 0.6. For the ‘inverted’ task (Fig. 6), the resting position was at the screen’s top and forward forces moved the Pac-Man icon downwards. Earlier experiments for monkey C used a single-degree-of-freedom load cell that measured only forward force. All experiments for monkey I and later experiments for monkey C employed a six-degree-of-freedom load cell. This was helpful in confirming, during the normal and inverted Pac-Man task, that forces were similar not only in the forward direction but also in off-axis directions.

Different force profiles were presented in interleaved trials. In each trial, a series of dots scrolled leftward on the screen at a constant speed (1,344 pixels per second). Subjects modulated Pac-Man’s position to intercept the dots, for which they received a juice or water reward (based on preference). Thus, the shape of the scrolling dot path determined the temporal force profile that the monkey needed to apply to the handle to obtain reward. We trained subjects to generate static, step, ramp, and sinusoidal forces over a range of amplitudes and frequencies. We define a ‘condition’ as a particular target force profile (for example, a 2 Hz sinusoid) that was presented on many ‘trials’, each a repetition of the same profile. Each condition included a ‘lead-in’ and ‘lead-out’ period: a static profile of 1 second (0.75 seconds for Monkey I) attached to the beginning and end of the target profile. Trials lasted 2.25–6 seconds, depending on the particular force profile. Juice was given throughout the trial, so long as Pac-Man successfully intercepted the dots, with a large ‘bonus’ reward given at the end of the trial. The reward schedule was designed to be encouraging: greater accuracy resulted in more frequent rewards (every few dots) and a larger bonus at the end of the trial. At high-frequencies, even slight errors in response phase (or latency) translate into a large vertical error, between Pac-Man’s height and dot height. To avoid discouraging failures, we tolerated small errors in the response phase at high frequencies. For example, if the target profile was a 3-Hz sinusoid, it was considered acceptable if the monkey generated a sinusoid of the correct amplitude and frequency but that led the target by 100 ms. To enact this tolerance, the target dots sped up or slowed down to match his phase. The magnitude of this phase correction was scaled with the target frequency and was capped at ±3 pixels per frame. To discourage inappropriate strategies (for example, moving randomly or holding in the middle with the goal of intercepting some dots), a trial was aborted if too many dots were missed (the criterion number was tailored for each condition). These criteria led to excellent performance on the vast majority of trials. Aborted trials were not analyzed, nor were trials where the generated force had an unusual profile that could not be aligned with other trials (and thus could not be included in trial averaging).

### Surgical Procedures and Intracortical Recordings

After initial training, we performed a sterile surgery to implant head restraints and recording chambers, positioned to allow access to premotor cortex (PMd), where response properties are very similar to those in surface M1. All recordings were made in the right hemisphere. Chamber positioning was guided by structural magnetic resonance imaging prior to implantation. We used intracortical microstimulation to confirm that recordings were from the arm region of motor cortex (biphasic pulses, cathodal leading, 250 ms pulse-width delivered at 333 Hz for a total duration of 50 ms). Microstimulation typically evoked contractions of the shoulder and upper arm muscles, including the *deltoid* and *triceps*. Current thresholds ranged between 20 and 200 *μ*A depending on the location and depth of stimulation (this wide range of current thresholds is expected, as we recorded in both M1 and PMd and from a variety of depths).

In Monkey C, the earliest recordings were made with 32-channel Plexon S-Probes, then the pre-production passive version of the primate-specific NeuroPixels probe, which allowed recording from 128 channels. Digitization (30 KHz) employed Blackrock Neural Signal Processors. Thereafter, and for all recordings in Monkey I, we employed the primate-optimized Neuropixels 1.0-NHP probes ^39^. Each such probe provided recordings from 384 channels, selected from 4416 total. Digitization (30 KHz) employed the standard system integrated within each Neuropixels probe, with data acquisition via connection to a PXIe system.

For Monkey C, the Neuropixels 1.0-NHP probe was held using a standard 0.25” dovetail mount rod with a custom adapter to mount it to a hydraulic drive (Narishige Inc.). A 21 gauge blunt guide tube, 25 mm in length, was held using a custom fixture and placed over the desired recording location. The dura was then penetrated with a tungsten electrode (FHC, size E), which was bent at 27 mm to prevent the tip from inserting further than 2 mm past the end of the guide tube. The tungsten electrode was inserted manually via forceps, once or several times, as necessary, which also provided feedback on the depth and difficulty of penetrating the dura. The Neuropixels 1.0-NHP probe was then aligned using the Narishige tower XY stage, lowered into the guide tube, and carefully monitored to ensure that the tip of the probe was aligned with the small dural opening created by the tungsten electrode.

For Monkey I, the Neuropixels 1.0-NHP probe was held using a custom fixture mounted to a linear rail bearing (IKO Inc.). This apparatus was designed to enable close packing of many probes, and to solve the challenge of precisely targeting structures deep in the brain. The linear rail was mounted in a custom 3D printed base, mounted directly to the recording chamber. The geometry of the 3D printed base component determined the insertion trajectories and prevented mechanical interference between probes and the chamber. This base also provided support for either sharp or blunt guide tubes, as required. In general, blunt guide tubes were preferred, but if necessary sharp guide tubes were sometimes used when the dura had become thicker and difficult to penetrate. The linear bearing was connected to a commercial drive system (NAN Inc.) via a ~50 mm long, 0.508 mm stainless steel wire, which provided rigid connection between the NAN drive electrode mount and the Neuropixels probe mounted on the rail bearing, yet also allowed a small amount of misalignment between the drive axis and the insertion axis. This apparatus simplified the procedure of using many probes in a small space, and avoided relying on commercial drives to provide the mechanical rigidity required to safely insert a delicate probe. Additional details on the custom hardware is provided in the Neuropixels 1.0-NHP user wiki ^39^.

### Spike Sorting and Unit Inclusion

Recordings were made at tip depths ranging from 5.6 mm to 12.1 mm relative to the dural surface (note that recording sites spanned multiple mm above the tip). Digitized voltages were spike-sorted using KiloSort 2.5. Sessions were analyzed only if probe drift was minimal. Both well-isolated single units and high-quality multi-units were included in analysis, after restriction based on several criteria. To be included in analysis, a unit had to be stable throughout the full session, had to have an average rate (across all times and conditions) > 1 Hz, and had to pass a threshold in terms of our ability to accurately estimate its modulation. For each unit we computed a signal-to-noise ratio (SNR). Signal was the modulation of the mean rate, assessed as its standard deviation across time and conditions. Noise was estimated as the average standard error of that mean (standard error computed across trials, then averaged across time and conditions). The ratio of these two numbers is high if the mean rate is both well-modulated and accurately estimated. We analyzed an isolation only if it had an SNR > 1.25. When considering a neuron’s firing rate, its across-time-and-condition range is considerably larger than its across-time-and- condition standard deviation. This is especially true if a neuron is particularly active in some situations but not others. Consequently, the above criterion (SNR > 1.25) yielded analyzed units that had firing-rate ranges considerably larger than the standard error with which that rate could be estimated. For example, this ratio was on average 31.9 (monkey C, 1257 analyzed units) and 12.6 (monkey I, 864 analyzed units) for the main datasets. An additional 477 units (average range-to-SEM ratio: 20.3) were analyzed for sessions where monkey C performed the normal-and-inverted Pac-Man task. (Monkey I performed both the basic task and the inverted task within the same sessions, so all recordings are from the same neurons). A further 217 units (average range-to-SEM ratio: 15.0) were analyzed for sessions where monkey C performed the task while being required to adapt to changes in gain.

### Trial Alignment and Averaging

Single-trial spike rasters, for a given neuron, were converted into a firing rate via convolution with a 25-ms Gaussian kernel. One analysis (Supp. Fig. 2) focused on single-trial responses, but most employed trial averaging to identify a reliable firing rate. To do so, trials for a given condition were aligned temporally on the moment when the target force profile ‘began’ – i.e. when the target force profile, specified by the dots, reached Pac-Man. Alignment brought the actual (generated) force profile closely into register across trials. However, because the actual force profile could sometimes slightly lead or lag the target force profile, some modest across-trial variability remained. We thus realigned each trial, by shifting it slightly in time to minimize the mean squared error (MSE) between the actual force and the target force profile. This ensured that trials were well-aligned in terms of the actual generated forces. Trials that could not be well-aligned despite searching over shifts from [−200, 200] ms were excluded from analysis. Alignment was applied to all conditions except the static forces, for which there was no need to do so. After alignment, the average firing rate (and its standard error) was simply the across-trial average (and standard error) of the filtered spikes.

### Data Preprocessing

PCA and other latent-factor estimation methods can be biased towards capturing the responses of a few high firing-rate neurons. For instance, a neuron whose firing-rate range is 10–100 Hz has more than 20 times the variance of a neuron whose firing-rate range is 10–30 Hz. Because neurons vary in their firing-rate range, any population will inevitably contain a subset of high-rate neurons, whose activity will dominate estimates of the latent factors. When estimating factors, this increases sampling error because factor-estimates are effectively based on fewer neurons. A common approach is thus to standardize responses such that each neuron has unit variance across time and conditions, allowing all neurons to contribute equally. This approach has the disadvantage that sampling error can be exacerbated for a different reason: low-firing rate neurons tend to have lower signal-to-noise ratios, and normalization increases the contribution of such neurons.

One wishes to strike a balance that minimizes these two effects. To do so, we employed a soft-normalization methods that we have consistently used across many studies (e.g. ^19,22^). Each neuron’s firing rate is soft-normalized by dividing by the firing-rate range plus a constant (5). To illustrate, consider that, when applied to the hypothetical neurons described above, soft-normalization shifts their effective firing rate range to be more equal: 0.095–0.95 and 0.286–0.857. The first neuron still contributes more variance, but only by about a factor of two. Soft-normalization thus allows factor estimation to be based more equally on all neurons, while still ensuring that neurons with low rates (and low SNRs) make smaller contributions.

### Motor-unit recordings

A motor unit is defined as a spinal motoneuron and the muscle fibers it uniquely innervates. When the motoneuron spikes, so do all its fibers, with essentially 100% reliability. Muscle-fiber spikes can be recorded intramuscularly. Each such spike corresponds, one-to-one, with the spike of a spinal motoneuron. A given muscle contains (depending on its size) on the order of 100 motor units. An advantage of recording motor-unit spikes (rather than bulk EMG) is that it facilitates direct comparisons between two neural populations: M1 neurons and spinal motoneurons. To aid such comparisons, motor-unit spiking was converted to firing-rate estimates using the same procedures as for M1 neurons (described above). All analyses of M1 population and motor unit populations thus paralleled one another. Motor-unit recordings were greatly aided by the isometric task we used, which provided unusually good recording stability, such that the voltage waveform, corresponding to a given motor-unit’s spike, remained consistent and sortable over the session.

As described previously ^28^, intramuscular EMG activity was recorded acutely using paired hook-wire electrodes (Natus Neurology, PN 019–475400). Electrodes were inserted ~1 cm into the muscle belly using 30 mm × 27 G needles. Needles were promptly removed and only the wires remained in the muscle during recording. Wires were thin (50 um diameter) and flexible and their presence in the muscle is typically not felt after insertion, allowing the task to be performed normally. Wires were removed at the end of the session. We employed several modifications to facilitate isolation of MU spikes. As originally manufactured, two wires protruded 2 mm and 5 mm from the end of each needle (thus ending 3 mm apart) with each wire insulated up to a 2 mm exposed end. We found that spike sorting benefited from including 4 wires per needle (i.e., combining two pairs in a single needle), with each pair having a differently modified geometry. Modifying each pair differently meant that they tended to be optimized for recording different MUs; one MU might be more prominent on one pair and a different MU more prominent on the other pair. Electrodes were thus modified as follows. The stripped ends of one pair were trimmed to 1 mm, with 1 mm of one wire and 8 mm of the second wire protruding from the needle’s end. The stripped ends of the second pair were trimmed to 0.5 mm, with 3.25 mm of one wire and 5.25 mm of the second wire protruding. Electrodes were hand-fabricated using a microscope (Zeiss), digital calipers, precision tweezers and knives. During experiments, EMG signals were recorded differentially from each pair of wires with the same length of stripped insulation; each insertion thus provided two active recording channels. Four insertions (closely spaced so that MUs were often recorded across many pairs) were employed, yielding eight total pairs.

Raw EMG voltages were amplified and analog filtered (band-pass 10 Hz - 10 kHz) with ISO-DAM 8A modules (World Precision Instruments), then digitized at 30 kHz with a neural signal processor (Blackrock Microsystems, Cerebus). EMG signals were digitally band-pass filtered online (50 Hz - 5 kHz) and saved. Offline, and prior to spike sorting, EMG signals were digitally filtered using a second-order 500 Hz high-pass Butterworth. Any low signal-to-noise channels were omitted from analyses. Motor unit (MU) spike times were extracted using a custom semi-automated algorithm. We adapted recent spike-sorting advances, including methods for resolving superimposed waveforms, documented extensively in ^28^. As with standard spike-sorting algorithms used for neural data, individual MU spikes were identified based on their match to a template: a canonical time-varying voltage across all simultaneously recorded channels. Spike templates were inferred using all the data across a given session. A distinctive feature of intramuscular records (compared to neural recordings) is very high signal-to-noise: peak-to-peak voltages are on the order of mV, rather than uV, and there is negligible thermal noise. At the same time, it is common for more than one MU to spike simultaneously, yielding a superposition of waveforms. This is relatively rare at low forces but can become common as forces increase. The motor-unit spike-sorting algorithm was thus tailored to detect not only voltages that corresponded to single MU spikes, but also those that resulted from superposition of multiple spikes. Detection of superposition was greatly aided by the multi-channel recordings; different units were prominent on different channels. We analyzed artificial spiking data with realistic properties (e.g. recruitment based on the actual force profiles we used, and waveforms based on actual recorded waveforms) to verify that spike-sorting was accurate in circumstances where the ground truth was known. Spike sorting criteria were extremely stringent, so that only very well isolated motor units were analyzed. Each recording session yielded 3–20 simultaneously recorded motor units, which were then pooled across sessions. This also involved pooling across muscles. For present analyses, such pooling is desirable because the task is performed using both the *triceps* and *anterior deltoid*, and stimulation of the recorded region of M1 activated these muscles. Consistent with the fact that these muscles are used together, the pooled motor-unit population still had low dimensionality.

We have previously documented that motor-unit recruitment is flexible ^28^. Recruitment reflects force profile, muscle length, and (when stimulating cortex) the precise stimulation site. The example motor-unit in Fig. 3 shows some flexibility: it becomes less active, for the same force, at higher frequencies. Motor-unit recruitment flexibility is one reason why the dimensionality of the motor-unit population is higher than one (the principal additional contribution being non-linearity – a motor unit typically remains silent below its force threshold). Yet while motor-unit flexibility exceeds traditional expectations, motor units never showed the extreme flexibility of M1 neurons. M1 neurons had a variety of correlations with force (positive, zero, and negative) and the sign and magnitude of these correlations often differed (for the same neuron) across conditions. This was not true of motor-units: if a motor-unit had a sufficiently low threshold (and thus was generally active across conditions) its activity consistently correlated positively with force.

### Estimating Control Strategies via Autoregressive Exogenous Input (ARX) Models

To estimate the control policy deployed in each condition, we modeled the force output on each trial using an autoregressive model with exogenous inputs (ARX): as described in Wasicko et al. ^76^. This model captures how force at each time point depends on its own recent history, the recent history of errors, and on an internal template. Specifically, we modeled the change in force at time t as:

(1) $$\dot{f}(t)=\alpha \dot{f}(t-1)+\sum_{k=1}^{n_{k}}\;\beta_{k}\cdot E(t-k)+\sum_{k=1}^{n_{f}}\;\sigma_{k}\cdot (\dot{f})(t-k),$$

where $\dot{f}(t-1)$ is the most recent derivative of force, E(t) is the force-tracking error, and $\left\langle \dot{f}\rangle (t\right)$ is the derivative of force averaged across all trials with the same force profile. The first of these terms yields dependence on recent history (providing smoothness). The second term provides dependence on error (the difference between force and target force). The third term acts as a template. For example, if the third term dominates, the predicted derivative of force can be entirely determined by the typical (trial-averaged) derivative. The parameters α, β, and σ were fit using ridge regression. Control policy distance (see below) was based on how well these parameters, when fit to one condition, accounted for data from another condition. Model hyperparameters (ridge penalty, number of parameters ($n_{e}$ and $n_{f}$)) were selected via cross validation on a held-out dataset (20% of the trials for each condition).

### Generalized Linear Models

We asked how well single-neuron (and single-motor unit) activity was explained via reference to force (Supp. Fig. 2). In addition to force, we also considered related variables that a feedback-controller might use. Specifically, we asked how well single-trial activity was accounted for by four explanatory variables: instantaneous force, error, and their derivatives. Analysis was performed at the level of single trials. Spikes were counted in 1 ms bins, and behavioral data were sampled at 1 kHz. The spike count was then fit via a Binomial Generalized Linear Model with a logit link function:

(2) $$p(s|\vec{x}(t))=\frac{1}{1\;+\;exp(-\beta \;\cdot \;\vec{x}(t))}$$

(3) $$\vec{x}(t)=[f(t+\tau ),\dot{f}(t+\tau ),e(t-\tau ),\dot{e}(t-\tau ),1]$$

(4) e(t)=f(t)-Target(t).

The lag, τ, was set to 80 ms for all units. We used this lag to reflect two assumptions. First, any dependence of neural activity on error (the visual difference between Pac-Man’s height and the dot height) will necessarily occur after the error itself. When visual events impact M1 activity (e.g. the response to target onset in a reaching task), they typically do so with a lag of 60–80 ms, hence this choice. Second, there will necessarily be a lag between neural activity and the downstream force it will cause. This lag is due both to axonal conduction and to the delay between electrical activation of muscle fibers and force generation. Estimates of this delay vary, but are typically in the 50–100 ms range. For the sake of simplicity we used a unified value of 80 ms for both lags (with opposite signs). Results were extremely similar across a reasonable range of choices (50–100 ms). Goodness of fit was calculated on a held out dataset using two metrics: bits per spike and R^2^. Bits per spike was calculated as,

(5) $$\frac{1}{ln(2)\sum_{i}^{N}y_{i}}\cdot \left(\sum_{i}^{N}y_{i}(\beta \cdot {\vec{x}}_{i}-\overset{‾}{y})-e^{\beta \cdot {\vec{x}}_{i}}+\overset{‾}{y}\right)$$

(6) $$\overset{‾}{y}=\frac{1}{N}\cdot \sum_{i}^{N}\;y_{i},$$

where N is the total number of spikes emitted by the neuron and $y_{i}$ denotes the $i^{\text{th}}$ spike of the neuron.

The second metric, *R*^2^, was calculated as the *R*^2^ between the predicted PSTH (after filtering and averaging over trials) and the empirical PSTH.

### Subspace Alignment Index

Activity for a given condition (or condition-group) occupies a set of dimensions – i.e. a subspace. To quantify the degree to which two conditions (or condition-groups) A and B share the same subspace, we employed the subspace alignment index ^29^:

(7) $$ai(A,B)=\frac{Tr\left(U_{B}^{⊤}\Sigma_{A}U_{B}\right)}{Tr\left(U_{A}^{⊤}\Sigma_{A}U_{A}\right)}.$$

Here, $U_{A}$ and $U_{B}$ are matrices whose columns contain the top k principal components computed for condition A and B respectively. The matrix $\Sigma_{A}$ is the covariance of the data during condition A. Thus the numerator is the variance from condition A captured by PCs from condition B, while the denominator is the variance from condition A captured in its own PCs (which by construction capture the maximum variance of any k-dimensional subspace). This index ranges from 0 (no alignment) to 1 (perfect alignment).

The value of the alignment index is typically robust across a range of values of k, but becomes sensitive at the extremes. At one extreme, if k equals the total number of recorded neurons, alignment will trivially be one for all comparisons. At the other extreme, if k=1, alignment may be low if the first dimension is not shared across conditions, even if other dimensions are. Given that we are interested in situations where alignment may sometimes become low, a conservative choice is to use a reasonably large value of k. We thus set k=15, as this exceeds the number of dimensions (roughly 10–12) typically assumed by the constrained-manifold hypothesis. Empirically, this value was well-suited to the present data, as it revealed both situations where activity was well-aligned and situations where it was not. That said, other reasonable choices produced nearly identical results (see below). For the motor-unit population, we set k=5 to be conservative in the opposite direction (to avoid overestimating alignment).

Another reasonable way of choosing k is as a percentage of the size of the recorded population. To explore this choice, we set k to be 3% of the total number of recorded neurons for that population (resulting in k≈30 for the M1 populations and k=3 for the motor-unit population). Results were nearly identical to those for the original analysis in Supp. Fig. 7. We also performed a control that matched both k and population-size for M1 and motor-unit populations (Supp. Fig. 8). The M1 population was downsampled (many times) to match the size of the motor-unit population, and we used the same value of k=3 for both (equivalent to 3% of neurons as described above). Alignment remained far lower for M1.

We performed two styles of alignment-index-based analyses: static and rolling. Static analyses considered activity over a large, static, epoch of time (the whole condition, or multiple similar conditions), which is how the alignment index has traditionally been computed. Static analyses employed a cross-validated approach (described below) that slightly improves the estimate of alignment by removing bias due to sampling error, while also symmetrizing the alignment metric. Rolling analyses considered a sliding window of activity in one of two conditions. For rolling analyses, we were less concerned with interpreting the exact magnitude, and more concerned with estimating its time-course. Furthermore, rolling analyses are intrinsically asymmetric (the sliding window applies to one condition but not the other). Rolling analyses thus use the standard non-cross-validated approach as in ^29^.

### Rolling Subspace Alignment Index

The rolling alignment index quantifies the degree to which activity, within a sliding window during a ‘test’ condition, is captured in a subspace computed from the full duration of activity during a ‘reference’ condition (Supp. Fig. 9 and Supp. Fig. 10). Let $A_{\text{test}}(t)$ correspond to data from within a sliding window of the test condition, from t-500ms to time t. Let $B_{\text{ref}}$ correspond to data from the full duration of the reference condition. The rolling alignment, at time t, was then

(8) $$ai\left(A_{\text{test}}(t),B_{\text{ret}}\right),$$

using the definition of the alignment index above. Confidence intervals (equivalent to standard errors of the mean) were computed using a bootstrap, by resampling across neurons.

### Cross-validated Subspace Alignment Index

Trial-by-trial noise can reduce the measured alignment between subspaces. Suppose conditions A and B were identical; i.e. evoked the same firing rates in all neurons. For a finite number of trials, the alignment index would still be slightly less than unity, because the principal components found for condition B will not be exactly identical to those for condition A, and the latter by definition capture maximal variance. This bias, though usually quite small, can cause alignment to be slightly underestimated. Given the nature of the hypotheses that were tested, we wanted to be careful not to underestimate alignment. We therefore computed a cross-validated subspace alignment index, adapted from the standard approach ^29^.

We constructed two independent sets of PSTHs for each condition by splitting trials into two non-overlapping groups. This resulted in two PSTHs per condition: one for group 1 trials and the other for group 2 trials. Using these splits, we constructed two separate subspaces for each condition group: $A^{1}$ and $A^{2}$. The cross-validated alignment index between condition groups A and B with (A≠B) was then computed as:

(9) $$\frac{ai(A^{1},B^{1})\;+\;ai(A^{2},B^{2})\;+\;ai(A^{1},B^{2})\;+\;ai(A^{2},B^{1})}{4(ai(A^{1},A^{2})\;+\;ai(A^{2},A^{1}))}+\frac{ai(B^{1},A^{1})\;+\;ai(B^{2},A^{2})\;+\;ai(B^{1},A^{2})\;+\;ai(B^{2},A^{1})}{4(ai(B^{1},B^{2})\;+\;ai(B^{2},B^{1}))},$$

where each term uses the standard alignment index described above. The cross-terms in the numerator average the empirical alignment across trial partitions: all combinations of A with B and B with A. The terms in the denominator provide an empirical estimate of the upper limit of alignment given sampling error; in the limit of infinite data, each of these terms would simply be unity.

### Sparse Component Analysis

A prediction of the flexible repertoire hypothesis is that population activity should involve distinct sets of factors (occupying distinct dimensions / subspaces) at different moments. Factors should thus be sparse in some ways (at a given moment, many factors may be inactive) but not others (factors may be reused across conditions if subskills are reused). Aspects of these predictions were confirmed by the alignment-based analyses in Supp. Fig. 9 and Supp. Fig. 10. For example, subspaces associated with slow increasing and slow decreasing ramps were sequentially reused during the 0.25 Hz sine condition but not the during the chirp, which instead reused the 1, 2, and 3 Hz subspaces. While these analyses demonstrate compositional reuse of subspaces, they relied on supervision. Specifically, they required an informed guess regarding which putative subskills were likely relevant when. Furthermore, although the alignment index has the advantage of simplicity (it is high when dimensions are reused and low when they are not), it does not allow one to directly inspect how the underlying factors evolve with time. This matters, because many of the predictions of the flexible-repertoire hypothesis pertain not only to when factors will be active, but to their time-course when they are active. For example, if a group of factors becomes active only for fast sines, that group should instantiate dynamics consistent with generating a fast sine, and should form a basis for decoding force during fast sines.

To estimate factors in an unsupervised manner, we applied Sparse Component Analysis ^47^ (SCA) to both M1 and motor-unit populations. Like PCA, SCA seeks to find latent factors that form a basis for the observed neural responses. That is, each neuron’s response should be well-approximated as a linear sum of the factors (e.g. Supp. Fig. 13). However, unlike PCA, SCA does not attempt to concentrate variance in the initial factors. The SCA cost function encourages maximization of variance captured across all factors, but not variance captured by (for example) the first factor. Additionally, the SCA cost function encourages the identified factors to be sparse if possible; the cost function is reduced if the basis includes factors that are active at some moments and not others. SCA can be viewed as a variant of PCA with an $L_{1}$ penalty applied to the projections to encourage sparsity.

SCA functions as a form of hypothesis-guided dimensionality reduction ^77^ that is ideally suited to the competing hypotheses being evaluated. Under the traditional constrained-manifold hypothesis, population activity should be accounted for by relatively few factors, most of which should be active across conditions (i.e. should be non-sparse). Because the SCA cost function is dominated by the goal of capturing variance, it cannot find sparse factors unless they form a natural basis set. Thus, under the constrained-manifold hypothesis, SCA should identify relatively few factors, most of which will be non-sparse. That SCA behaves this way for a low-dimensional non-sparse population response is confirmed by application to the motor-unit population (Supp. Fig. 14).

Under the flexible-repertoire hypothesis, accounting for the population response should require many factors, many of which will be sparsely active. Because the SCA cost-function encourages a sparse factor basis, if such a basis is not found, the flexible-repertoire hypothesis can be rejected. If a high-dimensional sparse basis is found, that would tend to support the flexible-repertoire hypothesis. One would then evaluate the behavior of the factors. There are many ways for factors to be sparse, and the flexible-repertoire hypothesis makes specific predictions (e.g. regarding dynamics during fast sines, as described above). Thus, a proper test of the flexible-repertoire hypothesis requires asking not only whether sparse factors exist, but also whether their activity, across time and condition, accords with predictions.

Two key hyperparameters govern the behavior of SCA: the total number of latent factors and the strength of the $L_{\text{1}}$ penalty, λ. Results were robust across a wide range of total factors. We set the number of total factors to be 3% of the number of recorded units, yielding 3 for the motor-unit population and ≈ 30 for the neural populations. This choice resulted in factors that captured the majority of population variance for both M1 and motor-unit populations. Additionally, the vast majority of individual factors (e.g. those plotted in Fig. 5 and Supp. Fig. 11) captured clear structure. If we increased the number of requested factors further, the additional identified factors tended to be low-variance (< 1%) and lack clear structure (i.e. they appeared to be dominated by sampling error). Thus, given the limitations imposed by population-size and trial-count, the number of factors whose time-course can be accurately estimated appears to be ≈ 30. This does not rule out the possibility that more factors could be accurately estimated with larger population sizes or higher trial-counts. At the other extreme, it was easy to avoid the regime where too few factors were requested, because this resulted in too little population variance being captured.

To test the robustness of our findings to the sparsity hyperparameter, we systematically varied λ. Doing so also acts as a further test of competing hypotheses. When λ is zero, the SCA cost function is more similar to that for PCA, and does not seek sparsity. At very large values of λ, the sparsity term dominates and reconstruction error will increase (the identified factors will be a poor basis). One can thus ask what occurs as λ is steadily increased. Different hypotheses make different predictions. Under the flexible-repertoire hypothesis, there exists a natural basis set where factors are somewhat sparse. Consequently, as λ increases, factor sparsity is predicted to increase before there is a meaningful rise in reconstruction error; the estimated factors will simply align better with the true sparse factors. This was indeed what occurred for both M1 populations (Supp. Fig. 15B,C). Under the constrained-manifold hypothesis, there does not exist a natural basis set of sparse factors. Thus, as λ increases, reconstruction error will rise before there is much change in sparsity. Put differently, increasing λ will increase sparsity only slightly, at the expense of a sizable increase in reconstruction error. This was indeed what occurred for the motor-unit population (Supp. Fig. 15A).

The analysis described above (and shown in Supp. Fig. 15) used two metrics of sparsity: excess kurtosis and latent sparsity. Excess kurtosis is defined as:

(10) $$EK=\sum_{i=1}^{k}\alpha_{i}\left(\frac{1}{T}\sum_{t=1}^{T}\;\frac{{\left(y_{i}(t)-\left\langle y_{i}\right\rangle \right)}^{4}}{\sigma_{i}}\right)-3,$$

where $y_{i}$ is the $i^{\text{th}}$ factor, $\sigma_{i}$ is its standard deviation, and $\alpha_{i}$ is the ratio of the variance explained by the $i^{\text{th}}$ factor relative to that explained by all k factors. Excess kurtosis measures how much higher the average kurtosis of the latent factors is than expected from a Gaussian distribution. A high excess kurtosis indicates the data is more heavy-tailed (thus sparser) than expected under a Gaussian null model. Latent sparsity is defined as:

(11) $$LS=\frac{1}{T}\sum_{i=1}^{k}\left[\alpha_{i}\frac{\sum_{t=1}^{T}y_{i}(t{)}^{2}}{{\left(\sum_{t=1}^{T}\left|y_{i}(t)\right|\right)}^{2}}\right].$$

Latent sparsity also measures how sparse the latents are but is bounded between 0 (not sparse) and 1 (maximally sparse).

### Control Policy Distance

To quantify the similarity between control policies inferred from different conditions, we define a control policy distance metric based on generalization error. Intuitively, if two conditions share a similar control strategy, then a policy learned from one should perform well when applied to data from the other.

Let $\pi_{i}=\pi \left(.;\alpha_{i},\beta_{i},\sigma_{i}\right)$ denote the control policy inferred from condition i, parameterized by the fitted autoregressive (α), feedback (β), and feedforward weights (σ) as described in Equation (1). Let $f^{a_{j}}\left(t\right)$ be the force generated for condition a on trial j and let ${\overset{ˆ}{f}}_{b}^{a_{j}}\left(t\right)$ be the predicted force trajectory for condition a on trial j using the control policy from condition b. The latter is obtained by integrating the predicted force derivative generated by $\pi_{a}$ over the all timesteps. We define the control policy distance between conditions a and b as:

(12) $$CP\left(a,b\right)=\frac{1}{2}\left(\frac{\sum_{j}\;\sum_{t}\;{\left(f^{a_{j}}\left(t\right)-{\overset{ˆ}{f}}_{b}^{a_{j}}\left(t\right)\right)}^{2}}{\sum_{j}\;\sum_{t}\;{\left(f^{a_{j}}\left(t\right)-{\overset{ˆ}{f}}_{a}^{a_{j}}\left(t\right)\right)}^{2}}\;+\;\frac{\sum_{j}\;\sum_{t}\;{\left(f^{b_{j}}\left(t\right)-{\overset{ˆ}{f}}_{a}^{b_{j}}\left(t\right)\right)}^{2}}{\sum_{j}\;\sum_{t}\;{\left(f^{b_{j}}\left(t\right)-{\overset{ˆ}{f}}_{b}^{b_{j}}\left(t\right)\right)}^{2}}\right),$$

This metric thus reflects the relative decrease in prediction accuracy when applying policies across conditions, normalized by each policy’s performance on its own data. A value of 1 reflects perfect generalization, while larger values reflect poor transfer across conditions.

### Force Distance

Force distance was computed as the difference between the average force value,

(13) $$f_{d}(a,b)={\left\|\langle f\rangle_{a}-\langle f\rangle_{b}\right\|}^{2}.$$

### Directionality Distance

The directional distance between two conditions was computed as the difference between the average of the sign of their derivatives,

(14) $$D_{dir}(a,b)=\|\langle Sign(\overset{.}{f})\rangle_{a}-{\langle Sign(\overset{.}{f})\rangle_{b}\|}^{2}$$

### Neural Distance Prediction

We computed, for each condition, its activity centroid: the location, in full-dimensional space, of neural activity averaged over time. Any two conditions thus had a distance between them, which was small for some conditions and large for others. To assess the factors that determined centroid separation, we fit (squared) centroid distance as a linear function of the three behavioral distances above: control policy distance, force distance, and directionality distance (each also expressed as a squared distance). To aid interpretation of regression weights, we normalized each feature to have unit variance over the dataset.

### Exponential Fitting Procedure

To quantify the relationship between neural centroid distance and subspace alignment, we modeled their relationship using a logistic function of the form:

(15) $$y=\frac{1}{1+e^{a^{\prime }+b^{\prime }x}},$$

where y is the subspace alignment index, x is the (unsquared) centroid distance between two conditions, and $a^{\prime }$, and $b^{\prime }$ are fitted parameters.

To estimate this model, we applied a logit transform to the alignment index:

(16) $$Y_{t}=\log\left(\frac{1}{y\;+\;\epsilon }-1\right),$$

where ϵ is a small constant added to ensure the argument of the log is always positive. We then fit a linear model to the transformed data:

(17) $$Y_{t}=a+bx,$$

where a and b correspond to the intercept and slope, respectively.

After fitting in the transformed space, we inverted the transform to obtain the final exponential predictions in the original space:

(18) $$\overset{ˆ}{y}=\frac{1}{1\;+\;e^{a+bx}}.$$

The significance of the relationship was assessed using the *p*-value of the slope from the linear model. The overall goodness-of-fit was computed using the untransfomed alignment index and the nonlinear model predictions.

### Linear Dynamics System Fitting

To capture the low-dimensional dynamics of population activity during each condition (Supp. Fig. 12), we fit a linear dynamical system (LDS) model to neural data using Dynamic Mode Decomposition ^78^.

We first reduced neural activity to its top 10 principal components. This was done separately for each condition. We then fit a 10-dimensional LDS of the form:

(19) $${\dot{x}}_{t}=Ax_{t},$$

where $x_{t}$ is the projection of the neural state vector onto the top principal components at time t and A is a learned matrix describing the linear dynamics.

DMD estimates the best linear approximation of the dynamics, A, by relating consecutive datapoints:

(20) $$\left(X^{\prime }-X\right)=AX,$$

where X, and $X^{\prime }$ contain the state vector projections at times t and t+1, respectively. We computed A using the standard DMD solution:

(21) $$A=\left(X^{\prime }-X\right)X^{†},$$

where $X^{†}$ denotes the Moore-Penrose pseudoinverse of X.

Dimensionality was fixed at 10 to capture the dominant low-rank dynamics while avoiding overfitting. While 10 dimensions would clearly be insufficient (under the flexible-repertoire hypothesis) to capture global dynamics, the present analysis simply seeks to find a local linear approximation that fits each condition on its own. This respects the hypothesis that different conditions involve activity in different regions of state-space, with different local linear approximations to the dynamics.

### Analysis of Decoding Accuracy versus Encoding Strength

We used ridge regression to fit linear decoders to reconstruct force based on neural activity, using the model,

(22) $$y_{t}=w^{T}\cdot \vec{x_{t}},$$

where the solution is given by,

(23) $$w={\left(XX^{T}+\lambda I\right)}^{-1}XY^{T}$$

where λ, X, and Y are the ridge penalty, the matrix of neural (or motor unit) responses, and the vector containing the values of force across time and conditions.

We quantified two aspects of this fit: decoding accuracy and encoding strength. Decoding accuracy was the $R^{2}$ value of the fit to $y_{t}$, computed for held-out data (trials not used during training). Parameters were fit using firing-rate and force estimates from one partition, and generalization performance was tested for another partition. Encoding strength quantified the proportion of population variance, within $\vec{x_{t}}$, that is captured by the decoding axis w, computed as

(24) $$\frac{Var\left(w_{u}w_{u}^{T}X\right)}{Var(X)}.$$

where $w_{u}=w/\|w\|$. For a population of pure force-tuned neurons, both decoding accuracy and encoding strength would be near unity. However, it is also possible for decoding accuracy to be high while encoding strength is low. For example, this would occur in a population where force is only one of many signals reflected in single-neuron responses.

Estimates of decoding accuracy and encoding strength depend on λ. As λ increases, stronger regularization encourages greater encoding strength. Decoding accuracy will also tend to initially improve, because regularization improves generalization. However, if the true encoding strength is low (i.e. if force is a low-variance signal within the population) increasing values of λ will soon cause decoding accuracy to decline. This occurs because the estimated decoding dimension (which is encouraged by regularization to be high-variance) can no longer align with the true (low-variance) dimension that encodes force. At very high values of λ, decoding will necessarily be poor simply because the weights w are forced towards zero.

Some analyses (Supp. Fig. 16A) consider the full relationship, as λ increases, between decoding accuracy and encoding strength. We also summarized this relationship by identifying the ‘knee’ where accuracy began to fall as encoding strength increased (Supp. Fig. 16B,C). To do so we chose the largest value of λ where decoding accuracy remained above 80% of its maximal value. Doing so estimates the strongest that encoding strength can plausibly be, given that force decoding must be accurate.

The above analysis was applied to trial-averaged responses. This choice reduces the impact of sampling error, and thus allows decoding accuracy to potentially approach unity (which indeed it sometimes did) even if encoding strength is low. Analysis was repeated using the derivative of force as the target variable (Supp. Fig. 16D-F). We also repeated analysis for M1, after restricting the population to the most force-tuned neurons (Supp. Fig. 17).

### Neural Dissimilarity

We computed the neural dissimilarity of the response of the $i^{\mathrm{th}}$ neuron, across contexts a and b, using,

(25) $$D_{i}(a,b)=\frac{\sum_{t=0}^{T}{\left(r_{i}^{a}(t)-r_{i}^{b}(t)\right)}^{2}}{\left.\frac{1}{2}{\left(\sum_{t=0}^{T}\left(r_{i}^{a}(t)-{\overset{‾}{r}}_{i}^{a}\right)\right)}^{2}+\sum_{t=0}^{T}{\left(r_{i}^{b}(t)-{\overset{‾}{r}}_{i}^{b}\right)}^{2}\right)},$$

where $r_{i}^{a}$ and $r_{i}^{b}$ are the mean firing rate of neuron i in the two contexts, and t indexes across all times within a condition. A dissimilarity of zero indicates an identical mean response across contexts, while a value larger than 1 indicates that across context differences exceed the within-context variance of activity. Dissimilarity was computed per neuron and per force-profile, to respect the fact that a neuron’s response might differ little across contexts for some profiles, and a lot for others (this was indeed true for monkey I, who performed a larger variety of force profiles). Cumulative distributions considered dissimilarities for each neuron and force profile.

### Cross-context Similarity

Cross-context similarity is a population-level metric that quantifies the similarity of neural population activity between contexts a and b. It is closely related to one minus the Neural Dissimilarity metric, but operates on projections onto the PCs rather than on individual-neuron responses. We first performed PCA jointly on neural activity pooled across both contexts. We then projected activity from each context onto the top k PCs, yielding context-specific trajectories $x^{a}(t)$ and $x^{b}(t)$ in a shared k-dimensional subspace. We set k=30 to capture sufficient overall variance while still rejecting noise due to sampling error. Similar results were obtained across a range of reasonable choices. Similarity was then computed as:

(26) $$CS_{\left(a,b\right)}=1-\sum_{i=1}^{k}\alpha_{i}\frac{\sum_{t=0}^{T}{\left(x_{i}^{a}\left(t\right)-x_{i}^{b}\left(t\right)\right)}^{2}}{\left.\frac{1}{2}{\left(\sum_{t=0}^{T}\left(x_{i}^{a}\left(t\right)-{\overset{‾}{x}}_{i}^{a}\right)\right)}^{2}+\sum_{t=0}^{T}{\left(x_{i}^{b}\left(t\right)-{\overset{‾}{x}}_{i}^{b}\right)}^{2}\right)},\;\sum_{i=1}^{k}\alpha_{i}=1.$$

The ratio computes, for each PC, the normalized squared difference in activity. That difference is then weighted by $\alpha_{i}$, which is proportional to the variance captured by the $i^{\mathrm{th}}$ dimension, normalized to sum to unity for the number of PCs considered.

### Removing Translation Between Contexts

To determine how much of the observed cross-context difference in population structure could be explained by simple mean shifts (translations), we computed similarity in a manner that ignores differences in the overall mean across contexts:

(27) $$CS_{\left(a,b\right)}=1-\sum_{i=1}^{k}\alpha_{i}\frac{\sum_{t=0}^{T}{\left(\left(x_{i}^{a}(t)-{\overset{‾}{x}}_{i}^{a}\right)-\left(x_{i}^{b}(t)-{\overset{‾}{x}}_{i}^{b}\right)\right)}^{2}}{\left.\frac{1}{2}{\left(\sum_{t=0}^{T}\left(x_{i}^{a}(t)-{\overset{‾}{x}}_{i}^{a}\right)\right)}^{2}+\sum_{t=0}^{T}{\left(x_{i}^{b}(t)-{\overset{‾}{x}}_{i}^{b}\right)}^{2}\right)}.$$

Comparing the original cross-context similarity to the translation-removed cross-context similarity thus quantifies the extent to which cross-context differences reflect a uniform shift versus changes in the shape of the trajectory.

### Removing Rotation Between Contexts

After removing translation (see above), we asked to what degree similarity could be further increased by a rotation of the data within the PCs. Towards this end we solved the following Procrustes problem:

(28) $$A^{*}=\underset{A}{arg\;min}{\left\|{\vec{x}}^{A}-A{\vec{x}}^{b}\right\|}^{2}\;\text{subject}\;\text{to}\;A^{⊤}A=I,$$

where ${\vec{x}}^{a}$ and ${\vec{x}}^{b}$ are the demeaned population trajectories, projected onto the top PCs (k=30 as above) for contexts a and b, respectively. Solving this problem yields the optimal orthogonal transformation, $A^{*}$, that best aligns the trajectories of context b with those of context a. We then applied the optimal rotation $A^{*}$ to the trajectories from context b, and recomputed the cross-context similarity as previously described.

## Supplementary Material

Supplement 1

Supplement 2
