# Supplementary material for: Motor cortex flexibly deploys a high-dimensional repertoire of subskills
Source: bioRxiv. 2025 Sep 8:2025.09.07.674717. Preprint. [Version 1] doi: 10.1101/2025.09.07.674717 (PMC12439889; doi:10.1101/2025.09.07.674717)
Supplement: Supplement 2 [file NIHPP2025.09.07.674717v1-supplement-2.pdf]

bioRxiv preprint doi: https://doi.org/10.1101/2025.09.07.674717; this version posted September 8, 2025. The copyright holder for this preprint (which was not certified by peer review) is the author/funder, who has granted bioRxiv a license to display the preprint in perpetuity. It is made available under aCC-BY 4.0 International license.

Supplemental Figures

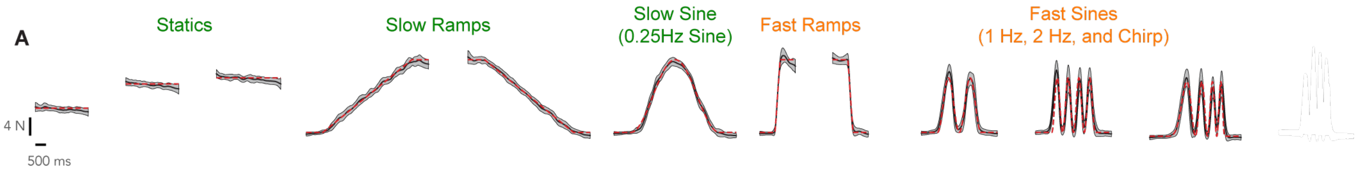

Supplementary Figure 1. Monkey I Behavior. (**A**) Same as for Fig. 2B but for Monkey I. Conditions are the same with two exceptions. First, the 2 Hz sine started and ended at the bottom, rather than the top. Second, Monkey I could not consistently perform the 3 Hz sine condition with high accuracy, and it was thus not included in his condition-set.

bioRxiv preprint doi: https://doi.org/10.1101/2025.09.07.674717; this version posted September 8, 2025. The copyright holder for this preprint (which was not certified by peer review) is the author/funder, who has granted bioRxiv a license to display the preprint in perpetuity. It is made available under aCC-BY 4.0 International license.

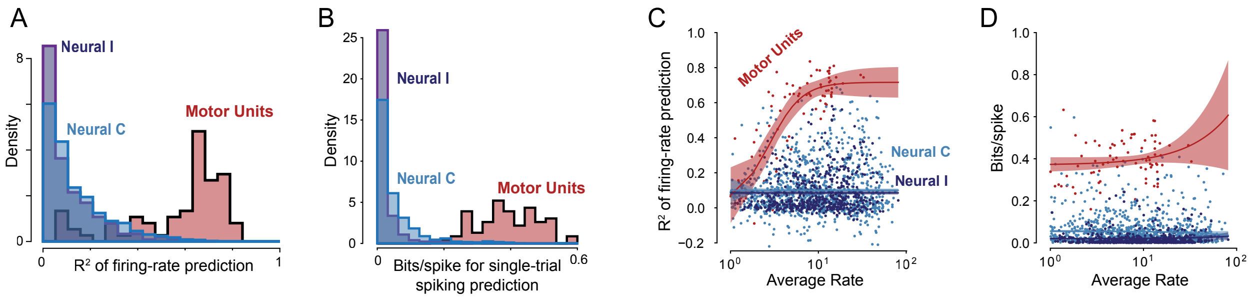

Supplementary Figure 2. Activity of single motor units, but not M1 neurons, strongly reflects force and related behavioral variables. We asked whether single-neuron spiking was a function of features a feedback controller might reasonably be expected to use: force, error, and their derivatives. To do so, we trained generalized linear models (GLMs) to predict spiking activity using these features. Model performance was evaluated on held-out trials. To quantify performance, we used both a cross-validated R^2 metric (computed between predicted and actual trial-averaged rates) and a single-trial bits/spike metric. (**A**) Distribution of R^2 values, shown separately for neural (M1) and motor-unit populations. Motor unit activity was well predicted by the GLMs, consistent with the role of motor units in force production. M1 neuron activity was predicted poorly. This agrees with what can be seen by inspection: it was rare for the rate of an M1 neuron to consistently encode force or its derivative. (**B**) Distribution of normalized bits/spike values across the same three populations in (A). Again, motor-unit activity was accounted for quite well, but M1-neuron activity was not. (**C**) A potential concern regarding the previous analysis (in panels A and B) is that differences in fits between M1 and motor-units might be secondary to differences in mean rate. To address this, for each neuron (blue) or motor unit (red), we plotted the R^2 of model fit is versus firing rate. Lines are fit using a Hill model; shaded regions denote 95% confidence intervals for model fit, computed via bootstrap. For a given rate range, motor-unit fits were typically much better. (**D**) Same as (C) but fit quality is assessed in terms of normalized bits/spike. Again, for a given rate range, motor-unit fits were typically much better. These analyses demonstrate that single-neuron firing rates in M1 rarely correlated well with force (or related variables). This does not imply that force cannot be decoded accurately from the M1 population response – it could, as will be documented below.

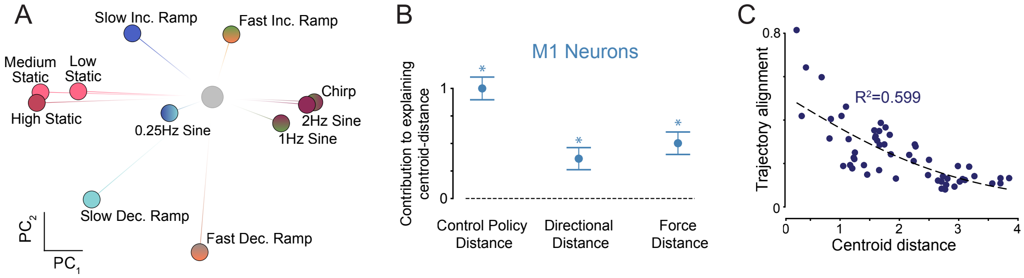

Supplementary Figure 3. Same as Fig. 4 but for Monkey I. Panel C of Fig. 4 already included results for monkey I; thus, that analysis is not repeated here. (**A**) Same analysis as in Fig. 4B. As for monkey C, centroids were well-separated. Aspects of centroid organization are also similar. For example, conditions requiring closed- versus open-loop control are well-separated. (**B**) Same analysis as in Fig. 4E. (**C**) Same analysis as in Fig. 4F. This analysis tests the flexible-repertoire prediction that, when two conditions use the same or similar subskills, centroid distance should be small and subspace alignment should be high. When they use very different subskills, centroid distance should be large and subspace alignment should be low. Consequently, centroid distance and subspace alignment should be negatively correlated when compared across condition pairs. To quantify the relationship between these two variables, we fit a logistic model by applying a logit transform and fitting a linear model to the transformed data (Methods). The significance of the slope was assessed in the transformed domain (linear-regression p-value), while the model fit (R^2) considered the final nonlinear predictions. Analysis was across all pairs of single conditions. Each dot corresponds to one condition pair and plots the alignment index between those conditions versus their centroid distance. Centroid-distance was computed in full-dimensional neural space. Solid line shows the fit. As for monkey C, there was a negative relationship between centroid distance and subspace alignment ($p < 10^{-5}$).

bioRxiv preprint doi: https://doi.org/10.1101/2025.09.07.674717; this version posted September 8, 2025. The copyright holder for this preprint (which was not certified by peer review) is the author/funder, who has granted bioRxiv a license to display the preprint in perpetuity. It is made available under aCC-BY 4.0 International license.

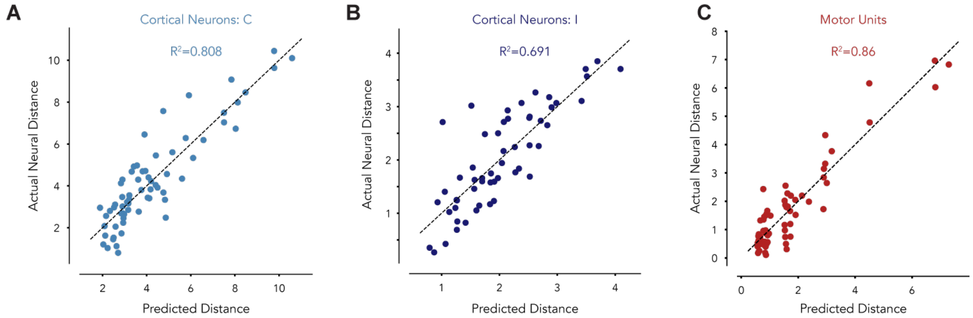

Supplementary Figure 4. Neural centroid distances were predicted well using a linear model. This analysis provides the basis for the analyses in Fig. 4E and Supp. Fig. 3B. Centroid distances were predicted based on three behavioral distances. These plots document those fits: they show actual versus predicted centroid distance for each pair of conditions. Fig. 4E and Supp. Fig. 3B document the contribution, to these fits, of the different behavioral distances. (**A**) Analysis for M1 centroids for Monkey C. Each dot corresponds to one pair of conditions, and plots the actual distance between their centroids versus the predicted distance. Actual distances were computed in the full-dimensional space after each neuron's rate was soft-normalized (see Methods). Distances thus have arbitrary units (rather than spikes/s). Predictions used a linear model that employed Force Distance, Directionality Distance, and Control Policy Distance as features (see Methods for details on how these were computed). (**B**) Same analysis for M1 centroids for Monkey I. (**C**) Same analysis for motor-unit centroids for Monkey C.

bioRxiv preprint doi: https://doi.org/10.1101/2025.09.07.674717; this version posted September 8, 2025. The copyright holder for this preprint (which was not certified by peer review) is the author/funder, who has granted bioRxiv a license to display the preprint in perpetuity. It is made available under aCC-BY 4.0 International license.

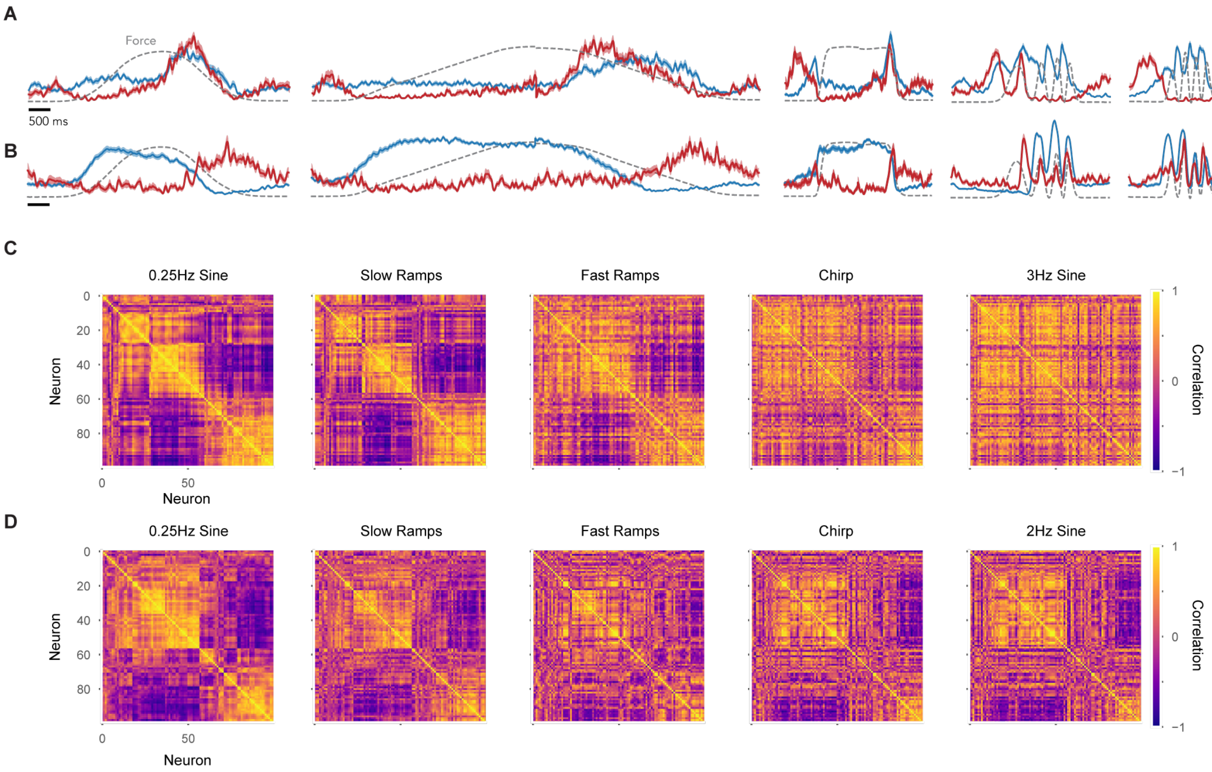

Supplementary Figure 5. Neuron-neuron correlations, when compared between conditions, are sometimes very similar and sometimes very different. A prediction of the flexible-repertoire hypothesis is that, when conditions differentially recruit subskills, neural activity will occupy non-identical sets of dimensions. A change in neural dimensions is equivalent to a change in neuron-neuron correlations. For example, suppose two neurons are both positively influenced by factor A, captured in dimension 1. Suppose that factor B, captured in dimension 2, has a negative influence on one neuron and a positive influence on the other. When a subskill primarily recruits factor A, the neurons will be positively correlated. When a subskill primarily recruits factor B, the neurons will be negatively correlated. Thus, before considering subspaces, we first examine neuron-neuron correlations. To aid visualization when viewing matrices of correlations, we restricted this analysis to the top 100 highest SNR neurons. (**A**) Average firing rate (envelopes show SE) for two example Monkey C cortical neurons, across various conditions. Dashed-black traces plot mean force. These neurons were positively correlated during low-frequency conditions (e.g. $r = 0.83$ at 0.25 Hz), but negatively correlated at high frequencies (e.g. -0.53 at 3 Hz). (**B**) Two additional example neurons from Monkey C showing the opposite pattern: $r = -0.74$ at 0.25 Hz and $r = 0.72$ at 3 Hz. These examples reflect a broader trend in the cortical population: neuron–neuron correlations shift relatively little between some pairs of conditions but rather a lot between others. In both example pairs, their correlation remained similar during the 0.25 Hz sine and the slow ramps, but reversed sign for the chirp. (**C**) Neuron-neuron correlation matrices for Monkey C. Neuron order was determined by a hierarchical clustering algorithm, which was used to highlight correlation structure during the 0.25 Hz sine condition. By sorting neurons to highlight structure, one can see when and whether this structure is maintained across other conditions. Correlations are shown for 5 conditions. For 'Slow Ramps' and 'Fast Ramps', we concatenated data across both directions (as illustrated in panel A). The correlation structure evident during the 0.25 Hz Sine is maintained during the Slow Ramps. This is consistent with a variety of other observations, made below. When comparing the 0.25 Hz sine with the fast ramps or the chirp, some broad structure is preserved but much is different (many rows/columns have changed their sign). Correlation structure is very different for the 3 Hz Sine. (**D**) Same analysis but for Monkey I. As above, aspects of neuron-neuron correlations are preserved across conditions (aspects of the block structure remain) while others change (many individual rows and columns change completely). As noted above, Monkey I did not perform the 3 Hz sine.

bioRxiv preprint doi: https://doi.org/10.1101/2025.09.07.674717; this version posted September 8, 2025. The copyright holder for this preprint (which was not certified by peer review) is the author/funder, who has granted bioRxiv a license to display the preprint in perpetuity. It is made available under aCC-BY 4.0 International license.

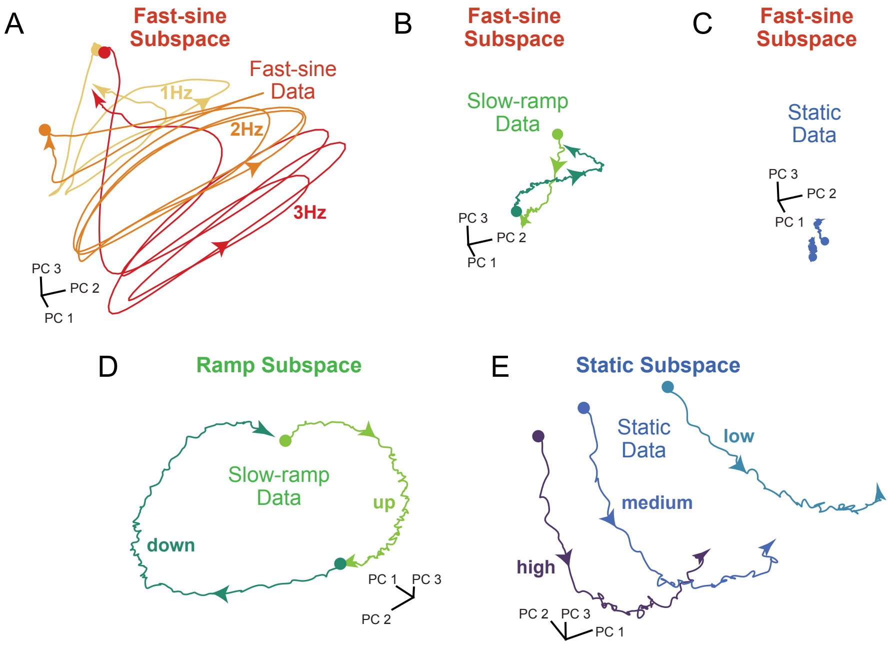

Supplementary Figure 6. Neural trajectories occupy different subspaces across conditions. The subspace-alignment metric, used in the main text, quantifies how well activity during one condition (or set of conditions) is captured by neural dimensions found for a different condition (or set of conditions). Here, instead of computing variance captured, we directly examine activity by plotting neural trajectories. Each panel plots the projection of neural population activity, for one set of conditions, into a subspace defined by the top three PCs computed from either the same conditions or from different conditions. All data are for Monkey C. (**A**) Neural trajectories for fast (1, 2, and 3 Hz) sine conditions, projected into a 'fast sine subspace' based on data from those same conditions. (**B**) Neural trajectories for the two slow ramps, projected into the fast sine subspace. Even though many neurons responded during the slow ramps, trajectories in this subspace are small (i.e. low variance) with unclear structure. (**C**) Neural trajectories for the three static conditions, projected into the fast sine subspace. Trajectories are barely visible; they are quite low-variance in this subspace. (**D**) Neural trajectories for the two slow ramps, projected into a 'ramp subspace' based on data from all ramp conditions (fast and slow). Trajectories are larger, with clearer structure, in this subspace. (**E**) Neural trajectories for the three static conditions, projected into a 'static subspace' based on data from those same conditions. Again, structure is present that was lost when trajectories were projected into the fast sine subspace.

bioRxiv preprint doi: https://doi.org/10.1101/2025.09.07.674717; this version posted September 8, 2025. The copyright holder for this preprint (which was not certified by peer review) is the author/funder, who has granted bioRxiv a license to display the preprint in perpetuity. It is made available under aCC-BY 4.0 International license.

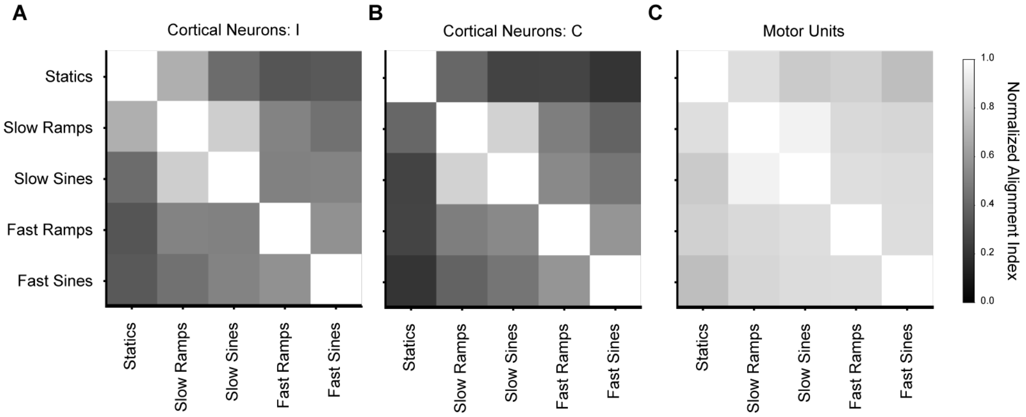

Supplementary Figure 7. Subspace alignment across condition-groups. Each matrix plots the alignment index for multiple comparisons. The alignment index ranges from zero to one, and asks how well activity for one group of conditions is captured by the top PCs from a different group of conditions, relative to how well activity would have been captured in its 'own' PCs. We used the following groups. Statics: low, medium and high statics; Slow Ramps: slow increasing and decreasing ramps; Slow Sine: 0.25 Hz sine; Fast Ramps: fast increasing and decreasing ramps; Fast Sines: 1, 2, and 3 Hz sines (3 Hz for monkey C only) and the chirp. Each condition-group involved a similar range of forces and (except for the statics) included both increasing and decreasing forces. Alignment was computed in a cross-validated fashion (using trial-partitions) and normalized to be unity when comparing a condition with itself (Methods). For M1, analysis was performed using a subspace dimensionality of 15 – i.e. slightly larger than the total dimensionality typically proposed under the constrained-manifold hypothesis. This choice is conservative; if the constrained-manifold hypothesis is correct, we wish to ensure dimensionality is not underestimated. For the motor-unit population, we wished to be conservative in the opposite direction, and thus chose a subspace dimensionality of five. To test sensitivity to these choices, analysis was rerun (for both M1 and motor-unit populations) using dimensionality equal to 3% of the size of the recorded population (≈ 30 for M1, 3 for motor-units). Results were extremely similar. (**A**) Analysis for the M1 population recorded from Monkey I. (**B**) Analysis for the M1 population recorded from Monkey C. (**C**) Analysis for the motor-unit population.

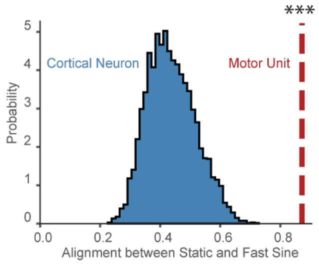

Supplementary Figure 8. Alignment Index Differences Are Not Due to Population Sizes. Results above demonstrate that alignment is consistently high for the motor-unit population, but becomes low, for some comparisons, for the M1 populations. Here we explore that result again after matching both the population size and the value of k (the number of dimensions used to compute alignment). We repeatedly sub-sampled the M1 population of Monkey C to match the size of his motor-unit population. We set $k = 3$ (i.e. 3% of the population size) for both M1 and motor units. (**A**) Distribution of alignment-index values when resampling the M1 population (blue), compared to the value for the motor-unit population (red line). The distribution of indices for M1 is significantly lower than, and non-overlapping with, that for the motor-unit population ($p < 0.001$). This analysis computed the alignment between the 'static' conditions (low, medium, and high) and the 'fast sines' (1, 2, and 3 Hz Sine conditions and the Chirp). Repeating analysis for all comparisons confirmed the original results; for comparisons where alignment was low in Supp. Fig. 7, alignment remained low after matching population size.

bioRxiv preprint doi: https://doi.org/10.1101/2025.09.07.674717; this version posted September 8, 2025. The copyright holder for this preprint (which was not certified by peer review) is the author/funder, who has granted bioRxiv a license to display the preprint in perpetuity. It is made available under aCC-BY 4.0 International license.

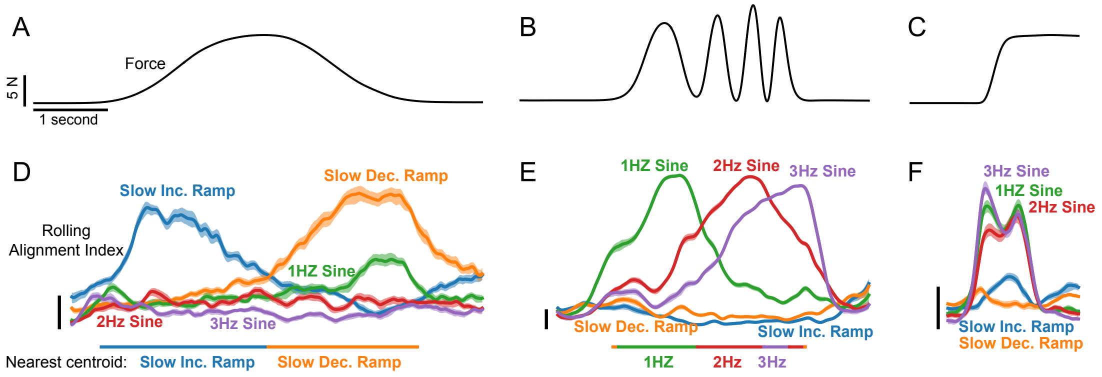

Supplementary Figure 9. Neural dimensions are reused in specific ways and at specific times (Monkey C). The analysis of subspace alignment above (main text and Supp. Fig. 7) reveals that the M1 population response sometimes reuses dimensions across groups of conditions. For example, alignment was higher when comparing the slow 0.25 Hz sine with slow ramps, and lower when comparing the slow 0.25 Hz sine with fast sines. The flexible-repertoire hypothesis predicts that this should happen for a specific reason: some conditions may reuse subskills compositionally. If so, there should be two forms of specificity: which subspaces align and when they align. Here we test this prediction by computing the alignment index, between various single conditions, as a function of time. We consider three 'test' conditions that might potentially involve compositionality: the 0.25 Hz sine, chirp, and fast-increasing ramp. For each, we ask how well the neural trajectory for that condition (in a 500 ms sliding window) is captured in subspaces for five 'reference' conditions: the three faster sines and two slow ramps. Subspaces for the reference conditions were computed across the full duration of the reference condition. We also computed the centroid for each reference condition (as in the main text), and determined if and when the trajectory for the test condition neared one of these centroids. (**A,B,C**) Average force, as a function of time, during the three test conditions. (**D**) Alignment when the test condition was a 0.25 Hz sine. Alignment indices are shown as a function of time. Each trace corresponds to one reference condition (as labeled). Envelopes denote 95% confidence intervals computed using a studentized bootstrap. Vertical scale bar denotes an alignment index of 0.1. The colored bar at the bottom indicates, across time, the reference centroid to which activity was closest. During the rising phase of the 0.25 Hz sine, activity occurs within the subspace used during the slow rising ramp, and comes nearest that centroid. During the falling phase, activity occurs within the subspace used during the slow falling ramp, and comes nearest that centroid. This effect is predicted if the 0.25 Hz sine condition reuses, in order, the subskills used during the slow rising and falling ramps. (**E**) Alignment when the test condition was the chirp. Activity aligns, in order, with subspaces for 1, 2, and 3 Hz sines. (**F**) Alignment when the test condition was a fast increasing ramp. Activity occurs, simultaneously, within subspaces for the 1, 2, and 3 Hz sines. This may potentially relate to the broad frequency content present in a rapid ramp. Activity did not near any single centroid, and thus no bar is shown at bottom.

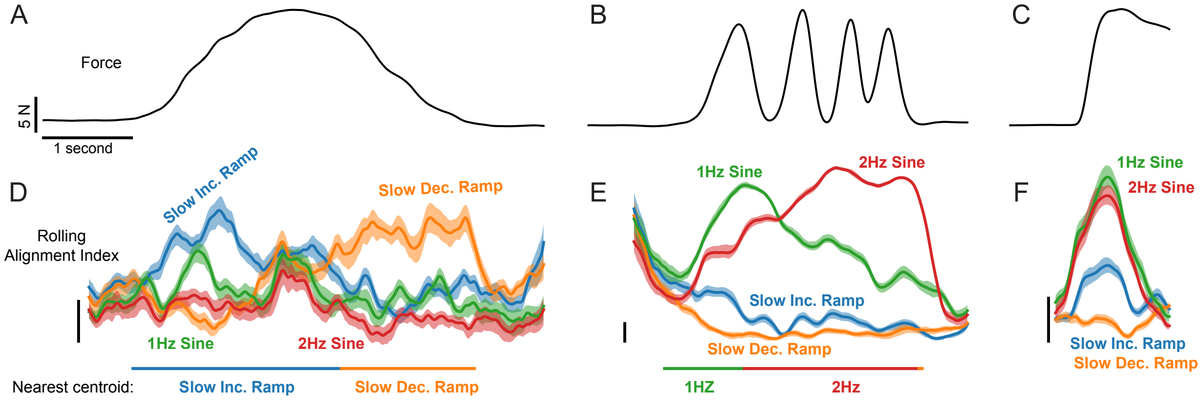

Supplementary Figure 10. Neural dimensions are reused in specific ways and at specific times (Monkey I). Same analysis as in Supplementary Figure 9, except for Monkey I. The 3 Hz sine is not used as a reference condition because Monkey I did not perform this condition.

bioRxiv preprint doi: https://doi.org/10.1101/2025.09.07.674717; this version posted September 8, 2025. The copyright holder for this preprint (which was not certified by peer review) is the author/funder, who has granted bioRxiv a license to display the preprint in perpetuity. It is made available under aCC-BY 4.0 International license.

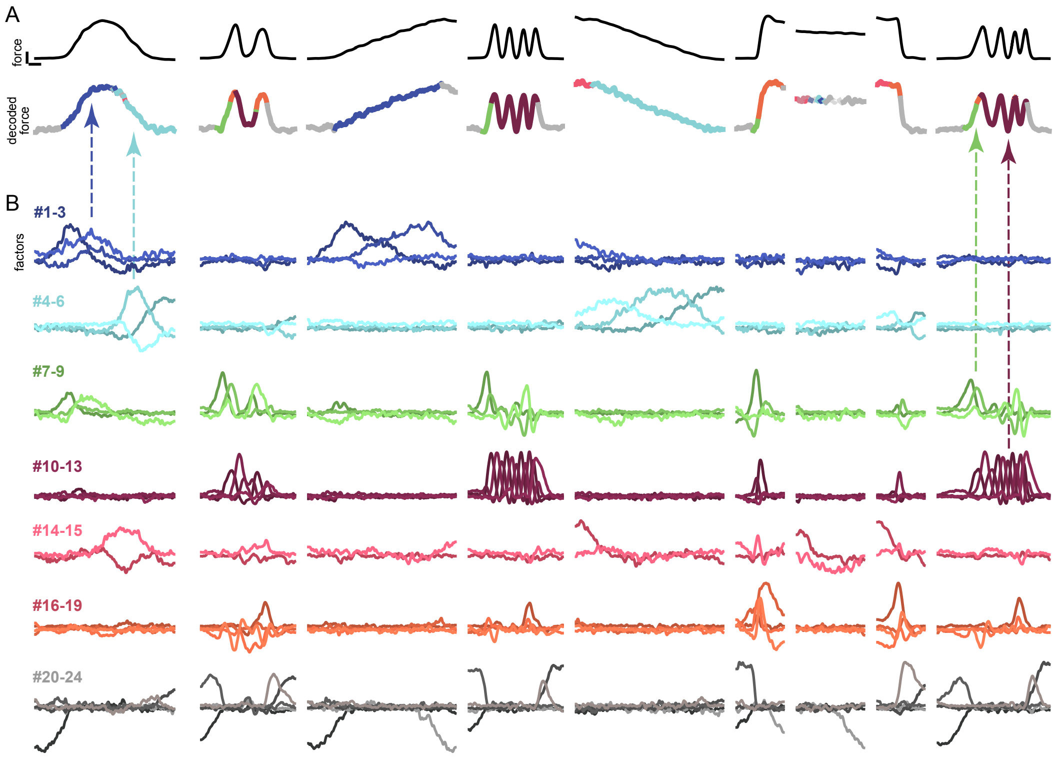

Supplementary Figure 11. Same as Fig. 5 but for Monkey I. Factors are grouped similarly, but not identically, to those for monkey C. It is anticipated that monkeys may differ somewhat in how they 'divide' the overall task into subskills. That said, most divisions, and instances of compositionality, were similar. For example, as for monkey C, the 0.25 Hz sine reuses, in order, the blue factor group (also used during the slow rising ramp) then the cyan factor group (also used during the slow falling ramp). This recapitulates, at the level of the factors themselves, the effect documented via subspace alignment in Supp. Fig. 10D. Similarly, the chirp uses the green factor group (also used during the 1 Hz sine) before the purple factor group (also used during the 2 Hz sine), in agreement with Supp. Fig. 10E.

bioRxiv preprint doi: https://doi.org/10.1101/2025.09.07.674717; this version posted September 8, 2025. The copyright holder for this preprint (which was not certified by peer review) is the author/funder, who has granted bioRxiv a license to display the preprint in perpetuity. It is made available under aCC-BY 4.0 International license.

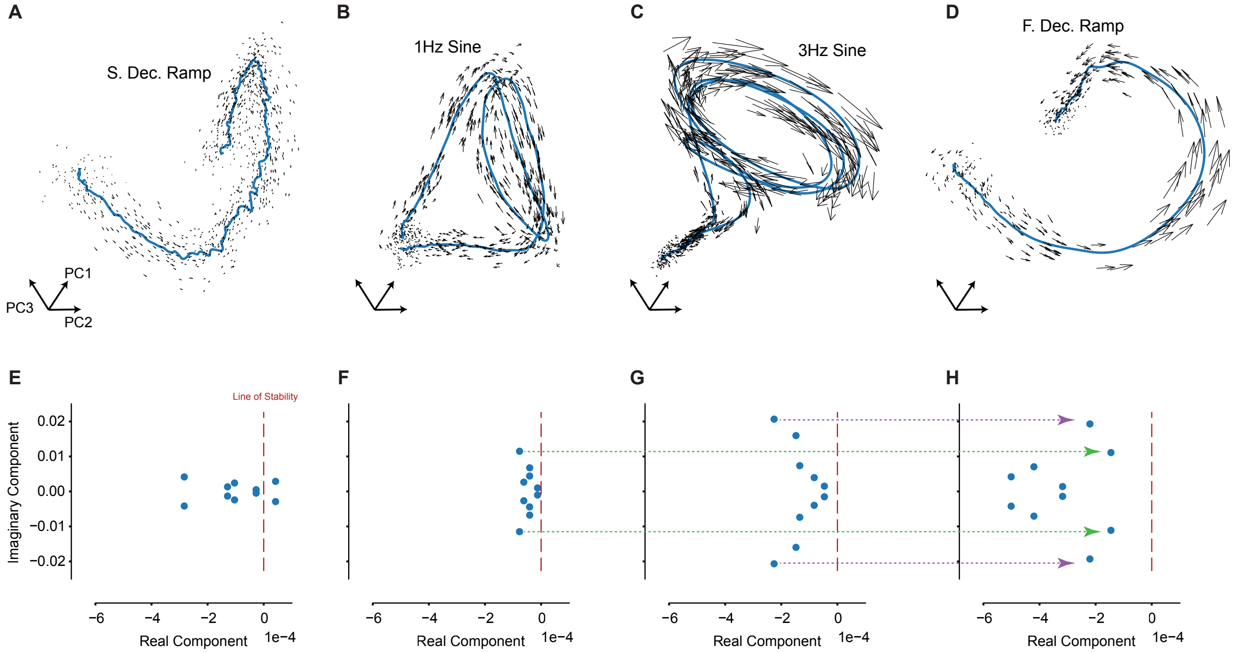

Supplementary Figure 12. Neural Dynamics Vary Across Conditions. The flexible-repertoire hypothesis posits that different subskills leverage different state-space locations and different dimensions (i.e. different factors). The purpose of doing so is to allow dynamics to differ across subskills while maintaining low trajectory tangling. A key prediction is thus that conditions should display a variety of neural dynamics, each appropriate to the condition in which they are deployed. We fit Linear Dynamical System (LDS) models to estimate the local flow-field for each condition (Methods). Each condition was fit separately, with fits based on activity in the dimensions used by that condition. If eigenvalues of the LDS models differ, then local dynamics differ. This approach is conservative; it is possible for dynamics to differ even when eigenvalues do not. Data are for monkey C, to allow comparison with factor-properties in Fig. 5. (**A**) Neural activity (blue) and the inferred flow-field (black arrows) for the slow decreasing ramp condition. The model was fit to the neural trajectory in the top 10 PCs. The plot shows projections, for both the trajectory and the flow-field, into the top 3 PCs. The flow-field is shown only locally near the trajectory. (**B-D**) Same as (A) but for other conditions. Note that each panel involves activity in a different global location (Fig. 4, Supp. Fig. 3) and in different dimensions (Supp. Fig. 6, Supp. Fig. 7). (**E**) Eigenvalue spectrum for the LDS model fit to the data in (A). The dashed red line denotes the maximum stable eigenvalue. (**F-H**) Same as (E) but for other conditions. Eigenvalue spectra reflect properties visible in the active factors (Fig. 5). For example, the slowly evolving cyan factor-group in Fig. 5 is active during the slow decreasing ramp, in agreement with the eigenvalues in panel E, which lack any fast oscillatory structure (the imaginary component is small). The oscillatory green factor-group is active during the 1 Hz sine, in agreement with the eigenvalues in panel F, which have a larger imaginary component. The higher-frequency oscillatory purple factor-group is active during the 3 Hz sine, in agreement with the higher-magnitude imaginary component in panel G. During the fast decreasing ramp, both green and purple factor groups are briefly active in Fig. 5. In agreement, the eigenvalue spectrum in panel H includes complex eigenvalue pairs that roughly match the higher frequencies from panels F and G (highlighted by arrows).

bioRxiv preprint doi: https://doi.org/10.1101/2025.09.07.674717; this version posted September 8, 2025. The copyright holder for this preprint (which was not certified by peer review) is the author/funder, who has granted bioRxiv a license to display the preprint in perpetuity. It is made available under aCC-BY 4.0 International license.

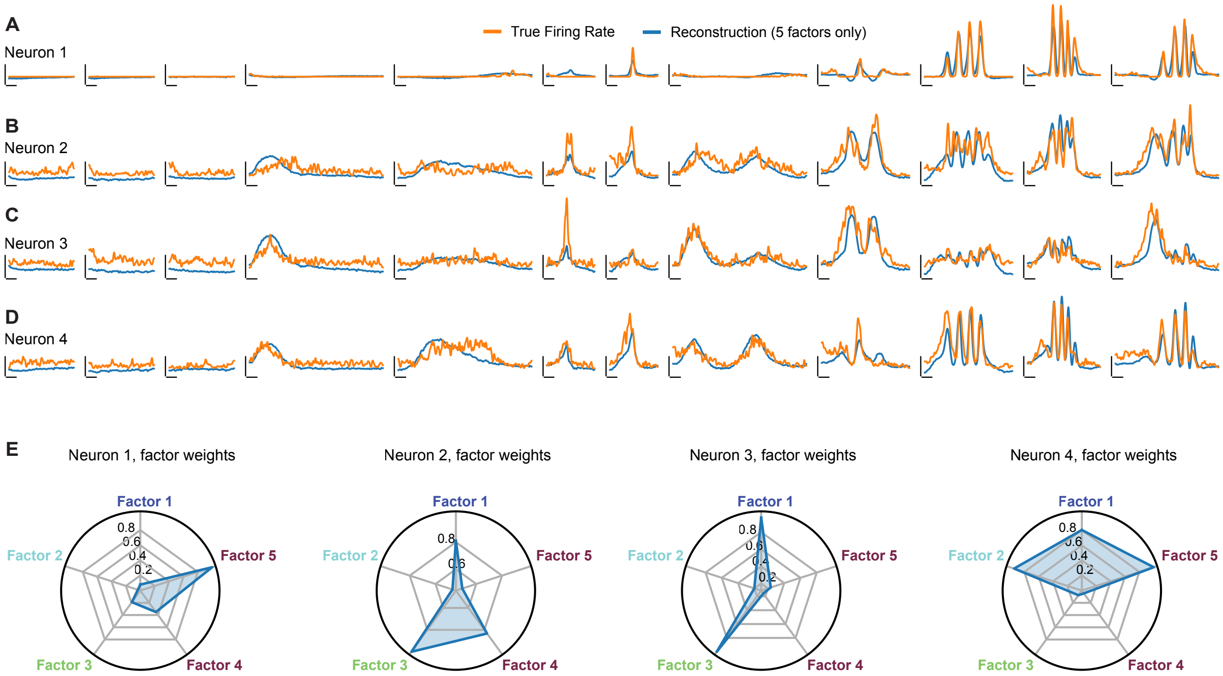

Supplementary Figure 13. Examples of how single-neuron firing rates reflect factors. Factors are population-level signals, yet also form a basis set for single-neuron responses. Here we illustrate this for four example neurons, by reconstructing each neuron's rate from five example factors. For ease of illustration, we intentionally chose example neurons that strongly reflected the same set of five factors. These factors were from the blue, cyan, green, and purple groups in Fig. 5. (**A**) Mean firing rate of one neuron (orange) and its reconstruction (blue) via a weighted sum of the five factors. (**B-D**) Same but for different example neurons. (**E**) Normalized contribution of each factor to the reconstructions above. Some neurons (e.g. neuron 1) had rates that primarily reflected one factor, and were thus quite sparsely active across conditions. Other neurons had rates that reflected multiple factors.

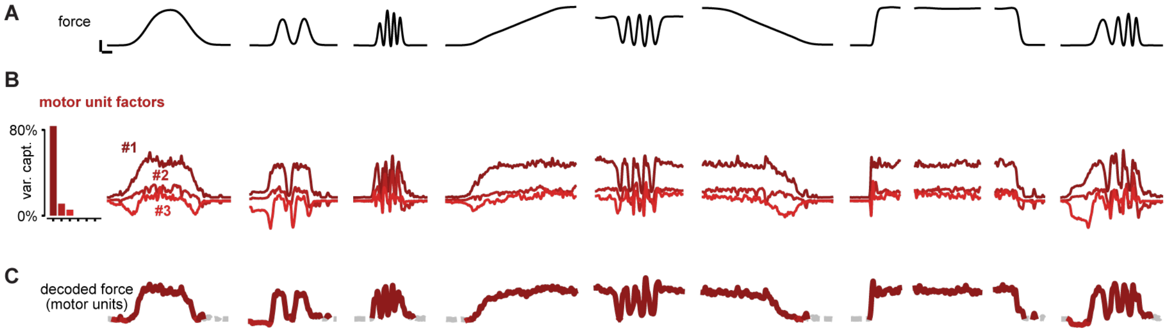

Supplementary Figure 14. SCA factors for the motor-unit population. Analysis parallels that for the M1 populations in Fig. 5 and Supp. Fig. 11. (**A**) Trial-averaged forces during different conditions. (**B**) Projection of motor-unit activity onto 3 SCA dimensions, yielding the factors. Unlike for M1, one factor dominated. Of the variance explained by SCA, $> 80\%$ is captured by the first factor (inset shows, for each factor, the percent of total captured variance that it accounted for). (**C**) Decoded force, based on a linear combination of the factors. The color of decoded-force trace indicates, at each moment, which factor made the largest contribution to decoding. Decoding relied almost entirely on the dominant factor. The trace is dashed gray at moments where no factor made a contribution (this occurred when the motor unit population was nearly silent).

bioRxiv preprint doi: https://doi.org/10.1101/2025.09.07.674717; this version posted September 8, 2025. The copyright holder for this preprint (which was not certified by peer review) is the author/funder, who has granted bioRxiv a license to display the preprint in perpetuity. It is made available under aCC-BY 4.0 International license.

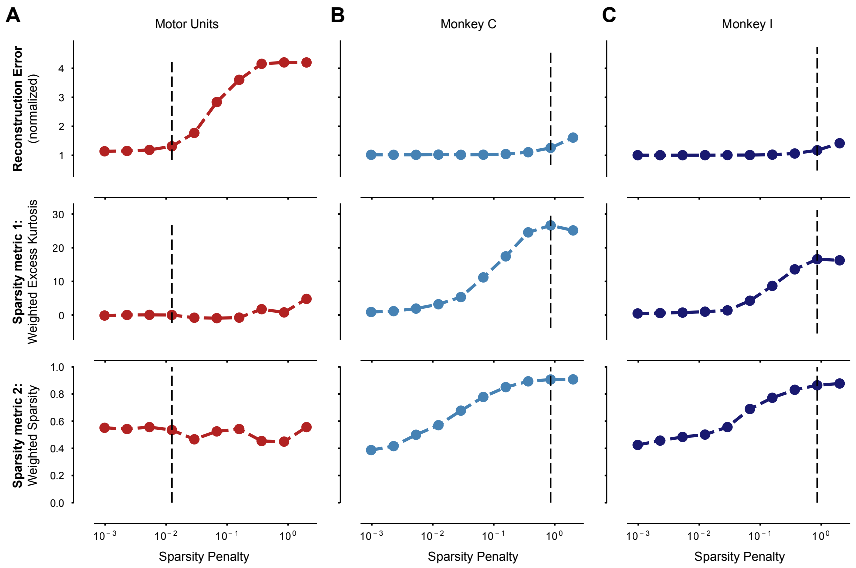

Supplementary Figure 15. For M1, but not the motor-units, SCA identified sparse factors and did so with little loss in overall variance captured. The SCA objective function includes a ‘sparsity penalty’ that encourages sparsity and a term that encourages variance captured (across all factors). This introduces a potential tradeoff between identifying factors that are sparse and identifying factors that capture variance. In situations where factors truly are sparse, this tradeoff should be minimal: there should be values of the sparsity penalty that identify sparse factors with little loss of variance captured (and thus minimal increase in reconstruction error when reconstructing individual-neuron responses from the factors). In situations where the factors are truly not sparse, this should not be true: increasing the sparsity penalty should produce a considerable increase in reconstruction error, and yet the estimated factors should still be relatively non-sparse. To explore, we swept the sparsity penalty and measured: reconstruction error (top row), excess kurtosis (a measure of sparseness, middle row) and weighted sparsity (a different measure of sparseness, bottom row). Reconstruction error was normalized by the PCA reconstruction error (PCA provides the minimum reconstruction error of all linear dimensionality reduction methods). Excess kurtosis asks how different the distribution of each factor’s values are from a Gaussian. Weighted sparsity was measured using the activity participation ratio. (**A**) Analysis for the motor units. Increasing the sparsity penalty caused a roughly four-fold increase in reconstruction error, but negligible increase in sparsity. Put differently, it was not possible to identify factors that were both sparse and provided a good basis for reconstructing motor-unit activity. The dashed black line corresponds to the sparsity regularization used to generate Supp. Fig. 14. This value was chosen so that the SCA factors explained $>$75% as much variance as the same number of principal components. (**B**) Same but for the M1 population recorded from Monkey C. As the sparsity penalty increased, factor sparsity increased considerably while reconstruction error remained low. Reconstruction error eventually increased, but only at high penalty values. Thus, it was relatively easy to identify factors that were both sparse and provided a good basis set for single-neuron responses. The dashed black line corresponds to the sparsity regularization used to generate Fig. 5. This value was chosen so that SCA factors explained $>$95% as much variance as the same number of principal components. Note that this choice was intentionally conservative: for the M1 population, sparse factors were found despite minimal increase in reconstruction error. This contrasts with the motor-unit population, where sparse factors were not found despite a larger increase in reconstruction error. (**C**) Same but for the M1 population recorded from Monkey I. The dashed black line corresponds to the sparsity regularization used to generate Supp. Fig. 11, and used the same criterion as in (B).

bioRxiv preprint doi: https://doi.org/10.1101/2025.09.07.674717; this version posted September 8, 2025. The copyright holder for this preprint (which was not certified by peer review) is the author/funder, who has granted bioRxiv a license to display the preprint in perpetuity. It is made available under aCC-BY 4.0 International license.

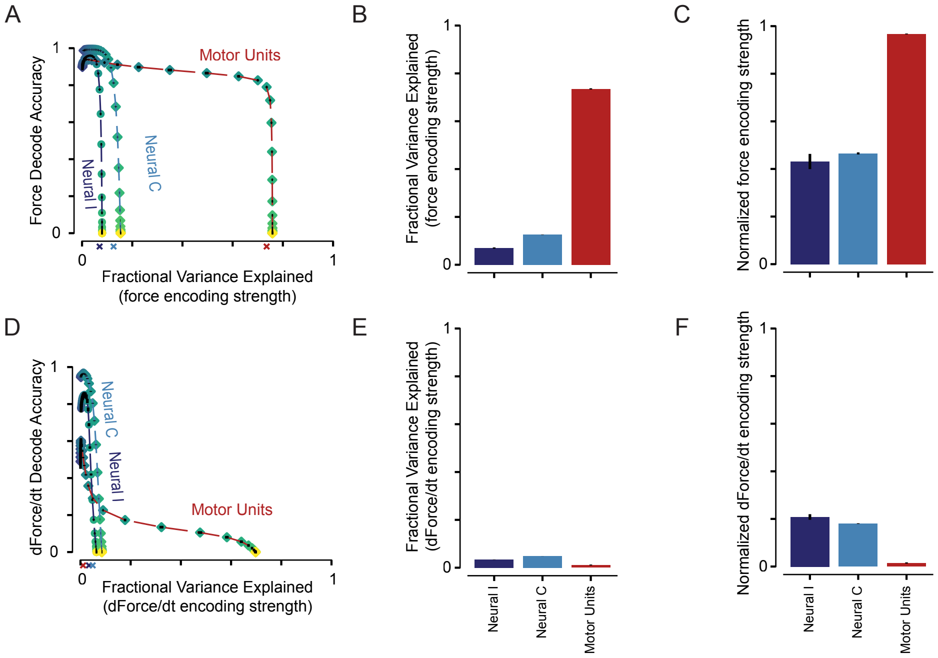

Supplementary Figure 16. Force is accurately decoded from both M1 and motor-unit populations, but the decoding dimension captures a large fraction of the population's response structure only for motor units. While the spiking activity of individual M1 neurons did not reliably reflect force, prior work argues that force (or, equivalently, commands for muscle activity) are encoded at the population level. The flexible-repertoire hypothesis similarly assumes that there exists a readout dimension where the projection of population activity provides descending commands. Thus, under both traditional hypotheses and the flexible-repertoire hypothesis, it should be possible to find a dimension where the projection of population activity yields accurate force decoding across all conditions. The Pac-Man task is very 'force-centric': many parameters, including Pac-Man's height, mirror force. Accurate force generation is also the fundamental goal of the task. Thus, under most traditional hypotheses, the force-decoding dimension should capture a large fraction of response structure. Put differently, there should exist a readout dimension where both decode accuracy (the force variance explained) and encoding strength (the neural response variance explained) are high. Under the flexible-repertoire hypothesis, the readout dimension is impacted by many factors (none consistently resembling force) and will thus explain only a small percentage of population response structure; encoding strength will never be high. We thus obtain the following prediction: encouraging the readout dimension to capture more response variance should impair decoding accuracy for M1 but not the motor units. To test this, we used ridge regression to predict force from population activity. Decode weights were learned based on trial-averaged responses from one partition of trials. Accuracy was then assessed when force was decoded based on trial-averaged responses from a second partition. Varying the ridge penalty (λ) allowed us to find a continuum of decoding dimensions that trade off decoding accuracy versus encoding strength. Analysis was repeated when decoding the derivative of force. (**A**) Force decode accuracy (R^2) as a function of encoding strength. Encoding strength was defined as the fraction of population variance explained by the decode dimension. Each point corresponds to a unique value of λ. For small values of λ, decode accuracy was high for both M1 populations and the motor units. As λ grew, so did encoding strength. For the M1 populations, decode accuracy declined almost immediately; it was not possible to find a dimension that both provided an accurate decode and captured considerable variance. In contrast, for the motor units, decode accuracy remained high across a range of λ, including values where encoding strength was quite high. Decode accuracy eventually declined for very high values of λ, simply because very strong regularization encouraged all weights to shrink towards zero. Colored x's indicate encoding strength when decoding accuracy was still at 80% of its maximum, which forms the basis for analysis in the next two panels. (**B**) To estimate the most neural variance that a force-decode dimension can capture, we measured force encoding strength at 80% of maximum decoding accuracy. This value is low for the M1 populations (blue) but high for motor units (red). (**C**) As in (B), but the variance explained by the decode dimension is normalized by the variance explained by the first PC of population activity. For the motor units, the value near one reflects the fact that the first principal component closely mirrors force. Values are lower ($\approx$0.4) for the M1 population, but are not tiny. The force-decoding dimension captures almost half as much variance as the first PC, which by definition captures the most variance of any dimension. Thus, force is a sizable signal in relative terms; the fact that it captures so little overall response variance is due to the high-dimensionality of the data, as expected given the proposal in Fig. 1A. (**D**) Same analysis as (A) except decoders were trained to predict the derivative of force (equivalent to the velocity of the Pac-Man cursor). Decoding accuracy was never high for the motor units. It was for the M1 populations, but accuracy declined swiftly at higher values of λ. (**E**) Same as (B) except when decoding the derivative of force. (**F**) Same as (C) except when decoding the derivative of force.

bioRxiv preprint doi: https://doi.org/10.1101/2025.09.07.674717; this version posted September 8, 2025. The copyright holder for this preprint (which was not certified by peer review) is the author/funder, who has granted bioRxiv a license to display the preprint in perpetuity. It is made available under aCC-BY 4.0 International license.

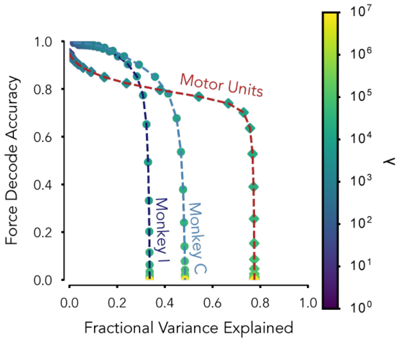

Supplementary Figure 17. After restricting analysis to M1 neurons with the strongest force tuning, force still explains less variance than for the motor unit population. A reasonable hypothesis is that there might exist a sizable subset of M1 neurons that encode force in a 'pure' fashion, as do motor units. Put differently, there might be a subset of neurons whose tuning is consistently aligned with the readout dimension that provides descending commands. Those neurons would consistently encode force, regardless of the currently engaged subskill. In contrast, the hypothesis in Fig. 1E supposes that descending commands are driven by different factors at different moments. Individual-neuron tuning reflects mixtures of those factors. Thus, pure force-tuning will be rare, and become rarer still when activity is examined across multiple putative subskills. To explore, we down-selected M1 populations to include only the 134 M1 neurons whose firing rates showed the highest absolute correlation with force. We chose 134 to match the size of the motor unit population, and because it was ∼10% of the the full M1 population. If there is a sizable subset of pure force-encoding neurons in M1, down-selection should restrict analysis to that subset, which should then behave much as the motor units. After down-selection, we repeated the decoding analysis described in Supp. Fig. 16 above. Plotting conventions are as in that figure. As expected, down-selected M1 populations had decoding dimensions that captured a larger fraction of population variance, relative to the full population. This can be seen in the plot by noting that decoding accuracy (vertical axis) remains high for larger values of variance explained (horizontal axis), relative to the original analysis in Supp. Fig. 16A. Yet even in the down-selected M1 population, the maximum proportion of population variance that could be explained (before decoding accuracy suffered) was only about half that for the motor-unit population. These results agree with our observation that the vast majority of neurons have activity that does not mirror force. Even when responses have a component that reflects force – such that force can be decoded – the force component is still mixed with other signals. Thus, down-selection enriches the strength of force encoding, but does not identify a subpopulation with pure motor-unit-like force encoding.

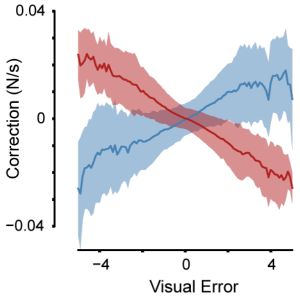

Supplementary Figure 18. Feedback corrections during normal and inverted Pac-Man for monkey I. Analysis parallels that for monkey C in Fig. 6A. Traces plot the average change in the derivative of force relative to baseline, 150 ms in the future, as a function of current visual error. Baseline was simply the mean derivative for the condition to which that trial belonged. Analysis collapses across all trials. Envelopes show standard deviations. Visual error is defined as the difference between Pac-Man's height and the height of the left-most portion of the dot-path. Red and blue traces correspond to normal and inverted subskills.

bioRxiv preprint doi: https://doi.org/10.1101/2025.09.07.674717; this version posted September 8, 2025. The copyright holder for this preprint (which was not certified by peer review) is the author/funder, who has granted bioRxiv a license to display the preprint in perpetuity. It is made available under aCC-BY 4.0 International license.

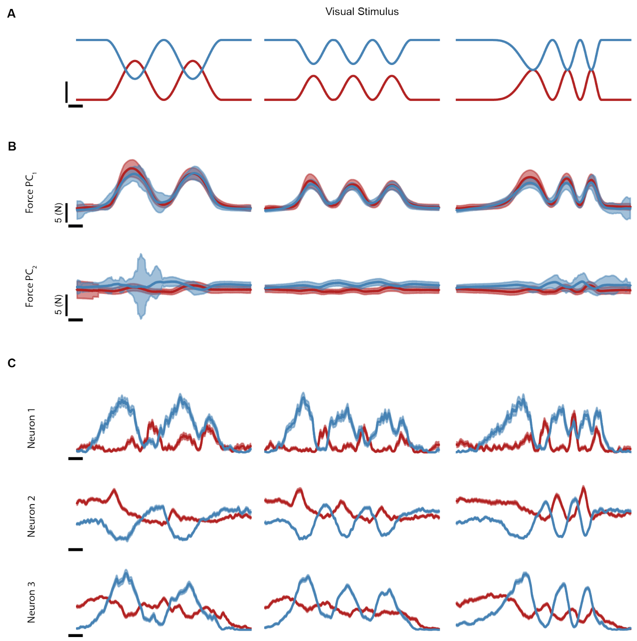

Supplementary Figure 19. Further analysis of behavior and additional example neural responses as monkey C performed normal (red) and inverted (blue) versions of the Pac-Man task. Analysis complements that in Fig. 6. Monkey C was trained on this two-subskill version of the task following the main experiments. Because this task is challenging and takes considerable time to learn, monkey C was trained on only a few conditions. (**A**) Traces show the visual dot paths, for both subskills, during three conditions. Within each condition, the different dot paths instructed the same force profile for both subskills. (**B**) As intended, and as shown in Fig. 6, similar forces were produced during both subskills. Here we examine that match by considering all three degrees of freedom measured by the 3-axis load cell. Doing so is important because it is conceivable that force could be well-matched only in the forward direction, and not in 'off axis' dimensions. We projected three-dimensional forces onto their first two PCs and computed the mean (solid line) and SD (envelopes) for each condition. The first PC is dominated by forward force, which is (as the task requires) well-matched across subskills. The second PC captures off-axis forces. These were small and differed only slightly between subskills. Thus, the two subskills involved very similar time-varying profiles of generated force, not only in the forward direction but overall. (**C**) Additional example responses from three neurons, complementing the example in Fig. 6B. Traces show the mean firing rate with SEM (envelopes). A 'pure' force-encoding neuron would show identical responses across subskills. This was observed only occasionally. These examples illustrate that neurons sometimes showed partially inverted responses, yet it was not the case that neural responses simply reflected the visual dot path. A variety of response changes, including amplitude and phase shifts, were observed. Examples for monkey I are shown in Supp. Fig. 20.

bioRxiv preprint doi: https://doi.org/10.1101/2025.09.07.674717; this version posted September 8, 2025. The copyright holder for this preprint (which was not certified by peer review) is the author/funder, who has granted bioRxiv a license to display the preprint in perpetuity. It is made available under aCC-BY 4.0 International license.

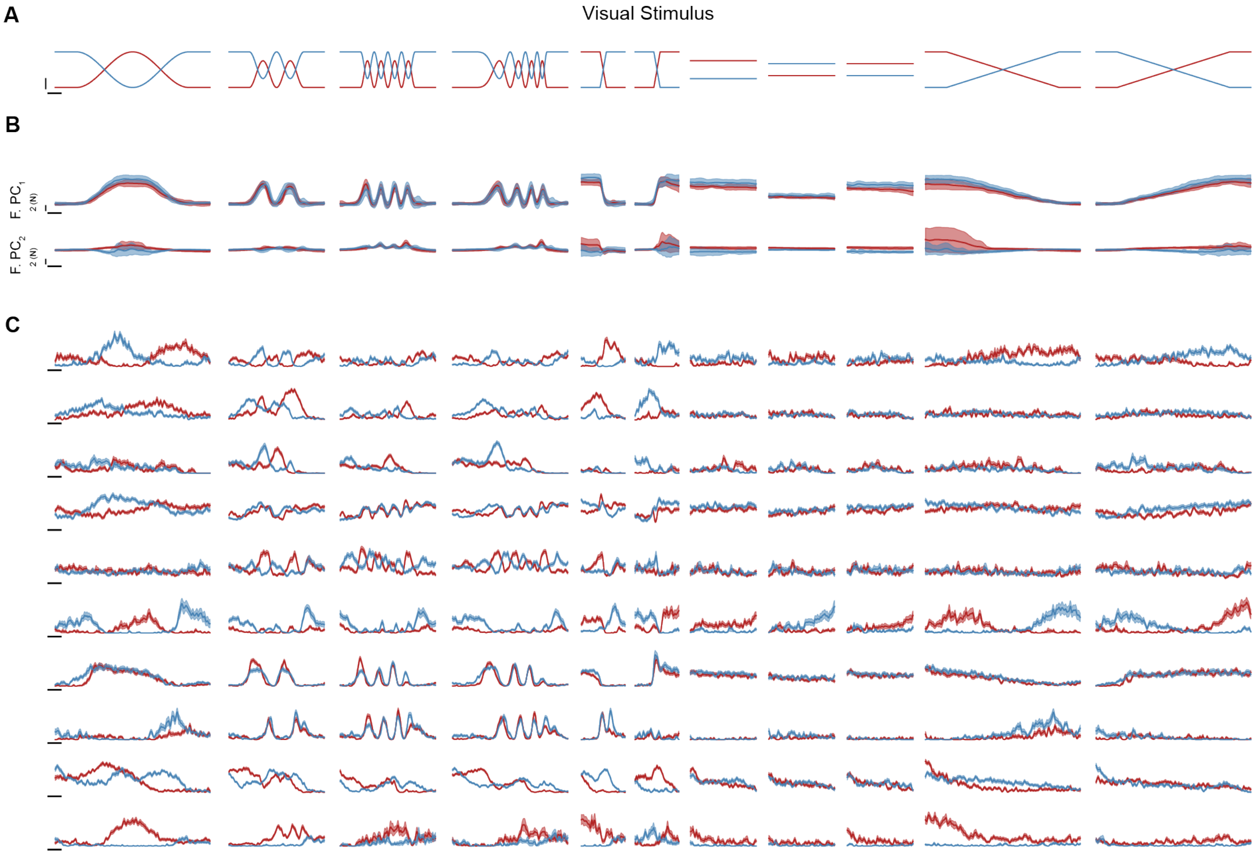

Supplementary Figure 20. Behavior and example neural responses as monkey I performed normal (red) and inverted (blue) versions of the Pac-Man task. Analysis is similar to that for monkey C in Supp. Fig. 19 above. Monkey I was trained on the two-subskill version of the task before the main experiments began, and became adept at doing so across a broad variety of conditions before recordings began. Hence the larger number of conditions shown here. Correspondingly, we also show a larger number of single-neuron examples. (**A**) Traces show the visual dot paths, for both subskills, for all conditions. Within each condition, the different dot paths instructed the same force profile. (**B**) As in Supp. Fig. 19, we examined the match in force by considering all three degrees of freedom. We projected three-dimensional forces onto their first two PCs and computed the mean (solid line) and standard deviation (envelopes) for each condition. The first PC principally captures forward force, which is (as the task requires) well-matched across subskills. The second PC captures off-axis forces. These were small and, for most conditions, differed only slightly between subskills. However, this was somewhat variable across conditions. Some conditions (especially the slow and fast falling ramps) involved off-axis forces for one subskill but not the other. A reasonable concern is that differences in neural responses across subskills might (trivially) reflect these differences in off-axis forces, rather than reflecting subskill per se. Fortunately, there were multiple conditions (e.g. the faster sinusoids) that had a nearly perfect match for both forward and off-axis forces. As can be seen via inspection of the next panel, neural responses often differed across subskills, and this was true across all conditions. Thus, the main source of such differences cannot be the occasional differences in off-axis forces. (**C**) Example neural responses. Traces show mean firing rate. Envelopes show SEMs. A wide variety of response profiles was observed. Occasionally, a neuron had consistently similar firing rates for both subskills (see third and fourth rows from bottom). Occasionally a neuron's response mostly inverted across conditions (see top row). Yet neither property was typical; responses typically differed in idiosyncratic ways that varied across conditions.

bioRxiv preprint doi: https://doi.org/10.1101/2025.09.07.674717; this version posted September 8, 2025. The copyright holder for this preprint (which was not certified by peer review) is the author/funder, who has granted bioRxiv a license to display the preprint in perpetuity. It is made available under aCC-BY 4.0 International license.

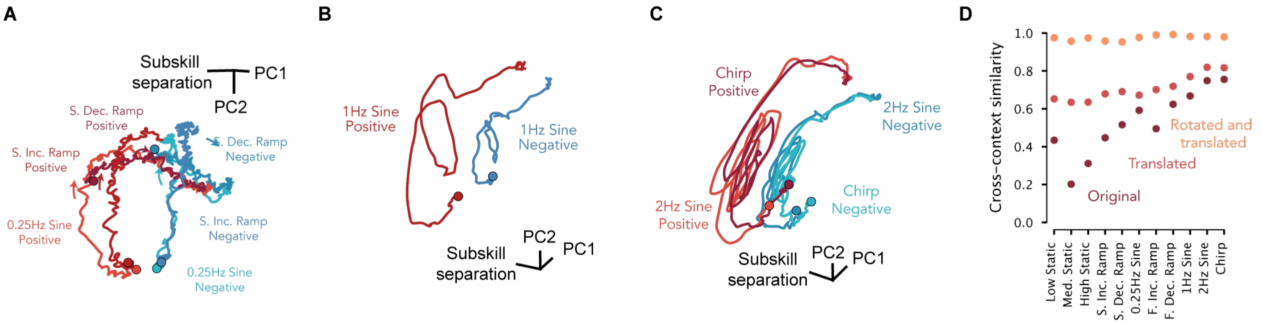

Supplementary Figure 21. Neural trajectories for normal and inverted subskills, for monkey I. Analysis parallels that for monkey C in Fig. 6C,D. Monkey I performed more conditions, and the degree to which neural activity reflected subskill was condition-dependent. At lower frequencies (e.g. the statics and the slow ramps), across-subskill differences in neural activity were quite large (indeed, larger than for monkey C). Across-subskill differences were smaller (but still sizable) for high-frequency forces. A speculation is that this may reflect the fact that slowly-changing forces rely heavily on closed-loop control, which is necessarily very different between normal and inverted subskills (the same error demands opposing responses). This may be less relevant when high frequencies demand open-loop (e.g. template-based) strategies. (**A**) State-space trajectories for both subskills (red and blue) during three low-frequency force profiles. Two axes were found via PCA (applied to these conditions only). The third axis was selected to capture the mean difference in neural activity between subskills (also for these conditions only). The two subskills involved similarly shaped neural trajectories. However, despite their similar shape, the trajectories were not the same. Trajectories occurred in different regions of state space, as evidence by the horizontal shift between red and blue traces. It was also the case (though it is hard to appreciate in three dimensions) that trajectories unfolded in somewhat different dimensions. (**B**) Same but for activity during the 1 Hz force profiles. (**B**) Same but for activity during 2 Hz and Chirp force profiles. (**D**) Same analysis as in Fig. 6D, but for Monkey I. Across-subskill differences in neural trajectories involved both a rotation and translation. As noted above, differences in neural trajectories between subskills were larger at lower frequencies. Once the translation and rotation were removed, trajectories were very similar.

bioRxiv preprint doi: https://doi.org/10.1101/2025.09.07.674717; this version posted September 8, 2025. The copyright holder for this preprint (which was not certified by peer review) is the author/funder, who has granted bioRxiv a license to display the preprint in perpetuity. It is made available under aCC-BY 4.0 International license.

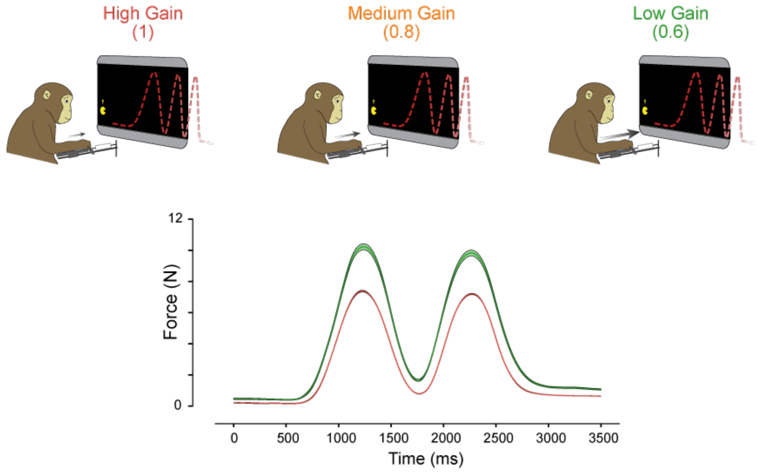

Supplementary Figure 22. Illustration of the task and behavior used to probe neural activity when force output must scale to compensate for changes in gain. Data are for monkey C. This experiment is intended (in part) as a control for the finding that M1 activity strongly reflects subskill when comparing normal and inverted versions of the task. The forces generated in normal and inverted Pac-Man were very similar, but small differences often remained. Might seeming subskill-specific aspects of activity instead reflect imperfectly matched forces? A natural control is to intentionally create larger differences in force, within subskill. Under the flexible-repertoire hypothesis, neural activity is predicted to change more as a result of changes in subskill than as a result of within-subskill changes in motor output. Because this prediction – activity is dominated by subskill – is fundamental to the flexible-repertoire hypothesis, testing it also provides an additional test of that hypotheses. To do so, we intentionally created sizable differences in force by altering the gain between the load-cell and the height of the Pac-Man cursor (top row). Gain was stepped down at discrete moments during the session and reached a low value of 0.6, such that 67% more force was needed to drive Pac-Man to the same height. Our expectation was that altering gain would produce rapid adaptation of the existing subskill, rather than learning of a new one. Consistent with this expectation, adaptation to the gain change was nearly instantaneous, and was largely complete even within the first trial. Forces during a gain of 0.6 were thus a scaled version of those during a gain of 1 (bottom panel, envelopes show SE). Under the flexible-repertoire hypothesis, only a very modest proportion of the population response reflects motor output, but a sizable proportion reflects subskill. Neural activity should thus differ less between gain changes than between normal versus inverted subskills, even though motor output changes more. This was indeed the case, as is documented below (Supp. Fig. 23, Supp. Fig. 24).

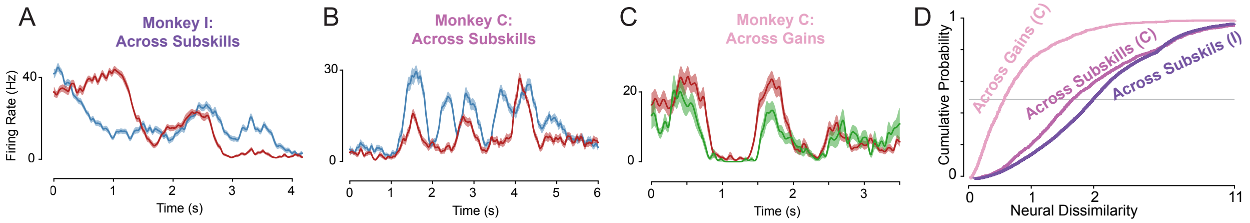

Supplementary Figure 23. Single-neuron responses differed much more across subskills (normal versus inverted) than across gains. Each example neuron was chosen to be representative of response differences for that experiment. Specifically, the neuron was chosen so that the normalized magnitude of the response difference was near the median. (**A**) Average firing rate (± SE) of a neuron recorded from monkey I during the Chirp Condition, for normal (red) and inverted (blue) subskills. (**B**) Same for a neuron recorded from monkey C during the 0.5 Hz sine (this panel repeated from the main text). (**C**) Same for a neuron recorded from monkey C when gain was high (red) and low (green). (**D**) Cumulative Density Function (CDF) of neural dissimilarity scores for monkey C (magenta) and I (purple) during the normal and inverted subskills, and for monkey C during the multi-gain experiment (gain of 1 versus 0.6). Dissimilarity was measured once for each neuron and force profile. Dissimilarity was higher across subskills, even though changes in motor output were smaller.

bioRxiv preprint doi: https://doi.org/10.1101/2025.09.07.674717; this version posted September 8, 2025. The copyright holder for this preprint (which was not certified by peer review) is the author/funder, who has granted bioRxiv a license to display the preprint in perpetuity. It is made available under aCC-BY 4.0 International license.

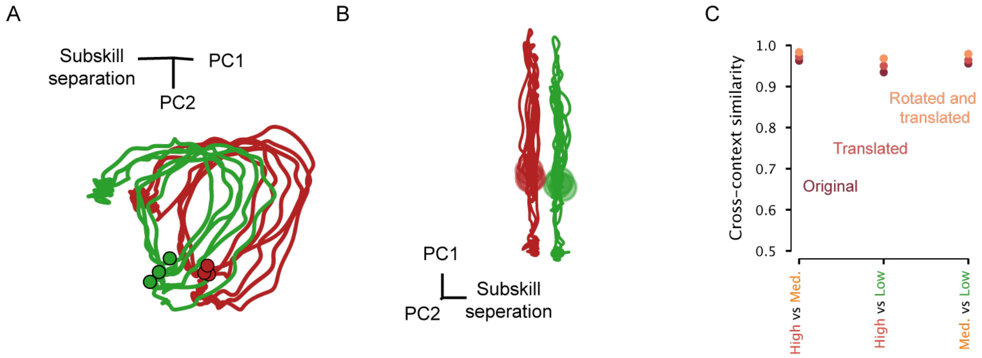

Supplementary Figure 24. The population response changed only modestly across changes in gain. This analysis parallels the analysis across subskills in Fig. 6C,D and Supp. Fig. 21. (**A**) The population response during a gain of 1 (red) and 0.6 (green) for all three conditions (different traces). (**B**) Same but from a different viewing angle, chosen specifically to maximize separation between the two gains. Separation was present but small. (**C**) Cross-context similarity of the population response, across gains, under different similarity maximizing transformations: no transformation (maroon), translation (red-orange), rotation and translation (orange). Similarity was already high before the transformations, and became only slightly higher after them.

Supplementary Movie 1. Video of the Pac-Man task being performed, and of motor-unit activity. The top panel is a black-and-white video of the screen, taken from inside the rig, as the Pac-Man task was performed by monkey C. The video was shot from above, hence the perspective. Video is from a session were multiple motor units were recorded. Motor-unit spikes appear in the bottom panel, which reproduces the experimenter's view of the recording system (Blackrock Microsystems), located outside the rig. The audio picks up on two sets of sounds: the spikes of a small handful of motor units (played on a loudspeaker outside the rig) and sound of the solenoid that controls juice delivery (also located outside the rig). Motor-unit spikes become more plentiful (and more motor-units begin to spike) in direct proportion to force, which is also reflected in the height of the Pac-Man icon. Yellow labels give a (rough) guess as to the behavioral strategy potentially used at different movements. Many of the features picked up on in the quantitative analyses of behavior, including small adjustments when forces changes slowly and swift commitment when force changes swiftly, can be seen by inspection.
